# Experimental Characterization of Steel and Concrete as Construction Materials: State-of-the-Art Methods and Advances Beyond Standardized Testing

**DOI:** 10.3390/ma19122498

**Published:** 2026-06-10

**Authors:** Marko Topalović, Vladimir Milovanović, Vladimir Dunić, Miroslav Živković, Snežana Vulović

**Affiliations:** 1 Institute for Information Technologies, University of Kragujevac, 34000 Kragujevac, Serbia; 2 Faculty of Engineering, University of Kragujevac, 34000 Kragujevac, Serbia; vladicka@kg.ac.rs (V.M.); dunic@kg.ac.rs (V.D.); miroslav.zivkovic@kg.ac.rs (M.Ž.)

**Keywords:** construction materials, steel, concrete, experimental testing, fatigue damage, experimental characterization, fracture mechanics, fracture toughness

## Abstract

Construction materials like steel and concrete have been used for thousands of years; however, their industrial-scale production began relatively recently in the 19th century. These materials are still being improved as the drive to build taller buildings, longer bridges, larger dams, and similar engineering marvels keeps pushing boundaries and requirements to previously unimaginable values. Yet, testing and characterization of construction materials that make all that progress possible are overshadowed in scientific literature by more trendy materials such as graphene, composites, nanomaterials, smart materials, and biomaterials. The objective of this review was to identify, collect, and systematically analyze recent papers in which the researchers performed experimental testing on construction materials to document how state-of-the-art experimental practice extends beyond what standardized protocols prescribe. This paper covers Uniaxial Tensile Testing (UT), Compact Tension C(T), Uniaxial Compression (UC), and Single Edge Notched Bending SEN(B), as they are the most commonly used and best-suited techniques for construction material analysis. State-of-the-art papers featuring these techniques were systematically gathered using AI-assisted literature discovery tools, and their contributions beyond ISO and ASTM standards were identified and summarized. Using this review, material scientists and engineers can quickly discover the most influential and relevant papers with the actual experimental data and can apply the testing procedures described in these papers in their laboratories so they can compare their results with the previously published measurements and make an engineering decision based on appropriate comparisons.

## 1. Introduction

**Figure 1:**
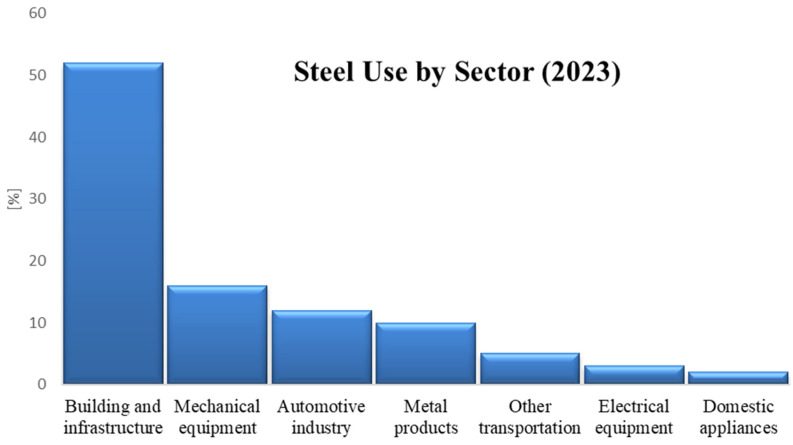
Distribution of total steel consumption in 2023.

The transition between Bronze Age and Iron Age occurred around 1200 BC (depending on the region). Erb-Satullo, in his review, pointed out inconclusive archaeological evidence of the early smelted iron, dating back as far as 2000 years BC [1]. According to the Erb-Satullo review, smelted iron artifacts appear more frequently in archaeological findings dating back to approximately 1200 BC and later [1]. The next significant technological advancement came in the form of Wootz steel, for which production began around 500 BC in South Asia, with the most prominent locations found in India and Sri Lanka [2,3]. This Wootz steel was a highly sought-after commodity in the ancient world and was the key component of Damascus steel, used to forge renowned weaponry between the 3rd and 18th centuries AD [4]. Due to the significantly reduced supply of Wootz ingots from India, in the 19th century, the technique of Damascus steel production was lost, so nowadays scientists use modern metallurgical methods to study and recreate this ancient technique [4]. In 1856, Henry Bessemer invented the Bessemer process, the first cheap method of mass-producing steel, which was crucial for the Second Industrial Revolution [5]. According to [5], the implementation of the Bessemer process and its subsequent improvements led to an increase in steel production from 15,000 tons in 1865 to 28 million tons in 1919. The rise in steel production continued throughout the 20th century, with no signs of slowing down. In 2023, global steel production reached 1.892 billion tons [6], with China’s 1.019 billion tons accounting for 53.9% of the global crude steel production [7]. According to the World Steel Association report “World Steel in Figures 2024” [8], Building and Infrastructure accounts for 52% of total steel consumption, followed by Mechanical Equipment at 16%, the Automotive Industry at 12%, and Metal Products at 10%. The remaining 10% is divided between Other Transport 5%, Electrical Equipment 3%, and finally, Domestic Appliances 2%. This distribution is illustrated in Figure 1.

Turning to Building and Infrastructure, at the moment, the bridge with the longest central span of 2023 m is the 1915 Çanakkale Bridge in Turkey, opened in 2022, with impressive 318 m high steel towers [9]. However, this bridge will not hold the title for long, as the Zhangjinggao Yangtze River Bridge in China (currently under construction) is projected to break six world records [10]. The main span will be 2300 m in length, with the South Tower (Jingjiang side) 350 m high and the North Tower (Zhangjiagang side) 359 m high [10]. For comparison, the Eiffel Tower is 330 m high [11]. However, unlike the 1915 Çanakkale Bridge towers, which are made entirely of steel, the Zhangjinggao Yangtze River Bridge towers are made using a steel–concrete composite design [10]. The same trend can be seen in the buildings as well. The tallest steel building (442 m tall), Willis Tower (also known as the Sears Tower), has defined the Chicago skyline since 1973 [12]. However, the tallest steel structure (tower) is Tokyo Skytree, opened in 2012, which is 634 m tall [13]. The tallest building overall today, the Burj Khalifa, which is 828 m tall, opened in 2010 [14]. But, unlike Willis Tower, the Burj Khalifa is mostly made of reinforced concrete with only the upper spire made of structural steel [14]. The Burj Khalifa will remain the tallest building until the 1 km tall Jeddah Tower (also known as the Burj Jeddah) is finished. The Jeddah Tower will feature the same building composition as the Burj Khalifa (primarily constructed using reinforced concrete, with only the upper spire made of structural steel). After discussing the largest steel bridges and buildings (with the additional comparison with concrete ones), attention now shifts to the biggest mobile structures ever made. The largest floating offshore structure currently operational is the Prelude FLNG (Floating Liquefied Natural Gas facility) [15]. Prelude FLNG is 488 m long, 74 m wide, and 105 m tall, with a displacement of 600,000 tons and made using more than 260,000 tons of steel, but it is not a ship in the sense because it is not self-propelled; instead, it is moored by a turret system anchored to seabed piles at the production site [15]. The largest actual ship, with a displacement of 365,000 tons and made using approximately 190,000 tons of steel, is Pioneering Spirit, which is a 382 m long and 124 m wide construction-crane catamaran [16]. To put the size of these vessels into perspective, RMS Titanic was 269 m long and 28 m wide, with a displacement of approximately 52,000 tons [17]. On land, the largest mobile structures (although not very mobile) are Bucket-Wheel Excavators. The world record holder is Bagger 293, standing 96 m high, 225 m long, and weighing 14,200 tons [18]. In comparison to this Behemoth, the Bucket-Wheel Excavator used in a Serbian coal mine, for which the Crawler travel gear was analyzed using the Finite Element Method (FEM), weighs merely 1200 tons [18]. As shown in [18], potential failures of Crawler travel gear parts would cause high financial losses, as replacement of damaged Bucket-Wheel Excavator parts must be executed on site, often in hard working conditions, so FEM analysis is used to identify critical areas that must be closely monitored. The areas with high stress concentration are prone to fatigue damage, a mechanism of progressive material degradation under cyclic loading, even when the maximum stress is below the static strength limit. An experimental comparison of mild-strength and high-strength construction steels [19] demonstrated that the use of high-strength steels increases the fatigue sensitivity of the construction components because the reduction in cross-section consequently leads to a decrease in flexural strength and hence an increased risk of buckling and fatigue failure. Thinner is not necessarily better. Although steel has approximately 10 times greater compressive strength than concrete, it is also 40 times more expensive and more prone to corrosion. Hence, using concrete for the construction of mega structures, such as the previously mentioned towers of the Zhangjinggao Yangtze River Bridge, Burj Khalifa, or Jeddah Tower, is economically more feasible and, at the same time, in terms of structural stability, more justified.

The history of the usage of concrete is somewhat different from the history of steel usage. Lime plaster, the earliest precursor to concrete, was used in the southern Levant from the 9th millennium BC [20]. Lime plaster was weak, slow hardening, and not hydraulic, but it was the first man-made binder. True concrete, in today’s terms, appeared several millennia later, in the 2nd century BC, in the form of Roman concrete (opus caementicium) [21]. As hydraulic concretes harden and gain strength through a chemical reaction with water, Roman concrete was often used for harbor construction, bridge building, and other waterworks. The legendary resilience of Roman concrete originates from the inclusion of pozzolanic ash into the mix that prevents cracks from spreading, and the hot mixing technique, where quicklime is mixed with water and aggregate at the same time, leading to significant heating up and creation of microcracks and lime clasts, which, when exposed to water, allow for self-healing of Roman concrete [21]. Unfortunately, the collapse of the Roman Empire (5th century AD) was accompanied by the decline of large-scale building projects, and without continuous practice and expertise transmitted through apprenticeship, the knowledge of Roman concrete production was gradually lost. Similar to Damascus steel [4], fascination with Roman concrete prompted scientists to use modern-day techniques to uncover its ancient secrets [21]. But many centuries passed before John Smeaton renewed interest in hydraulic lime in the 1750s [22]. In 1796, James Parker patented “Roman cement” [22]. The next milestone was set by Joseph Aspdin in 1824 when he patented Portland cement, a key component of modern concrete [22]. In 2022, global production of Portland cement reached 4.16 billion tons [23], with China’s 2.1 billion tons accounting for 50.5% of global Portland cement production [23]. The tallest concrete structures were already noted above, when discussing record-holding steel bridges and buildings, but the greatest concrete works are undoubtedly dams, and the largest of them all is the Three Gorges Dam on the Yangtze River in China [24]. The Three Gorges Dam, made using 27.2 million m^3^ of concrete and 463,000 tons of steel, is 2309.5 m long with a crest elevation of 185 m [24]. In comparison, Hydropower Plant Djerdap 1 (HPP Djerdap1) between Serbia and Romania on the Danube River is 1278 m long and 55 m high [25]. The FEM solver PAK (Program for Analysis of Constructions Kragujevac) [25] serves as a component of the Djerdap 1 Dam Monitoring System (DMS). The aging Djerdap 1 dam, built in the 1970s, requires close monitoring of the actual condition and behavior, such as stress levels, seepage patterns, and sediment accumulation, in order to ensure safe operation and optimum energy production. The PAK solver performs static, dynamic, and thermal analysis, as well as flow through porous media, providing a prediction of the state of the dam in the near future, forecasting problems before they occur, and facilitating preventive maintenance. The precise material properties are of utmost importance for accurate FEM analysis of dams, the same as mesh quality and boundary conditions. In order to keep up with the state-of-the-art trends in numerical modeling of material behavior and to provide the clients with the most accurate FEM simulations, the Phase-Field Damage Model was incorporated in the PAK code [26]. While phase-field damage modeling provided the motivation for this review, numerical modeling lies outside the scope of the present work, which focuses exclusively on experimental characterization.

Prior research by the present authors, spanning Bucket-Wheel Excavator analysis [18], comparative studies of construction steels [19], FEM dam simulations [25], and incorporation of a Phase-Field Damage Model into FEM code [26], revealed the absence of a comprehensive review of experimental procedures used for testing of construction materials. The significance of these materials is evident, now more than ever, yet the experimental research on these materials is eclipsed by more trendy materials like graphene, composites, nanomaterials, smart materials, and biomaterials. Despite the key role that steel and concrete, the most commonly used construction materials, play in infrastructure, energy systems, and heavy machinery applications, their experimental characterization remains scattered across a fragmented body of knowledge consisting of specialized industry standards, data from material vendors, and experimental results from limited or inconsistent tests. This paper consolidates scattered testing approaches into a structured, test-type-based classification of state-of-the-art experimental studies, explicitly documenting for each of the four covered test types the governing standards, the beyond-standard advances reported in the literature, and the reasons that drive researchers to deviate from standardized procedures.

Review papers are common across all scientific disciplines; however, reviews focused on the experimental testing of construction materials are exceptionally rare. The closest match to this paper that the authors could find is “A Review of Benchmark Experiments for the Validation of Peridynamics Models” published by Diehl, Prudhomme, and Lévesque [27]. This paper covers much wider material categories, which include both steel and concrete, but also aluminium, composites, glass, plastic, ice, and other materials, while performing a much narrower search using just Google Scholar and Web of Science with very specific keywords like “peridynamics + experiment”, “peridynamics + benchmark”, “peridynamics + computer graphics” and “peridynamics + visualization” [27]. It also studies a wider range of experimental procedures, which include Compaction Tension tests and Three-Point Bending tests, but also many more, which are focused on the impact mechanics, such as Taylor impact experiments, Ballistic impact test, the Kalthoff–Winkler experiment, and Split-Hopkinson Pressure Bar. With such a diverse, multi-focused approach, they missed a lot of state-of-the-art papers featuring experimental testing of construction materials. Another related paper was published by Volegov, Gribov, and Trusov in 2015, entitled “Damage and Fracture: Review of Experimental Studies” [28]. This paper covers a wide range of crystalline solids, with particular attention given to the study of fatigue failure of various metals and alloys. However, unlike [27], it lacks a clear classification based on experiments performed and a detailed comparison between reviewed studies. Also, concrete is not a crystalline solid; in fact, it is a composite material with an amorphous and disordered structure, so it is not covered in this review paper.

Unlike either of these works, the present review is organized around test type rather than material type or modeling framework, enabling direct comparison of methodological choices, instrumentation, and beyond-standard advances across studies that share the same experimental procedure but differ in material, application, or research objective.

Furthermore, review papers that deal with the experimental testing of concrete are extremely narrowly focused; take the paper by Tabrizikahou et al., “From experimental testing to computational modeling: A review of shape memory alloy fiber-reinforced concrete composites” [29], as an example. Ultra-high-performance reinforced concrete is state-of-the-art when it comes to this construction material [30], with countless more papers published on the same topic. Another cutting-edge approach is “smart concrete”, which uses nanoparticles to achieve self-sensing, self-healing, and self-adjusting concrete [31]. Essentially, using the 21st-century high-tech methods to achieve the same behavior that the Romans engineered centuries ago using ancient technology [21].

In this review, however, the papers that showcase experiments done on a wide range of steel grades are covered, from commonly used mild-strength steel to Ultra High Strength Steel (UHSS), as well as a wide range of concrete mixtures, from structural lightweight concrete (SLC) and normal strength concrete to Ultra-High Performance Concrete (UHPC).

The Experimental Methods segment of the paper is divided into four sections, with the first focused on uniaxial tensile testing (UT) using flat and cylindrical dog-bone specimens, covering the following topics:

- Measurement techniques and instrumentation;
- Standard flat specimens, mild and low-carbon steels;
- Standard flat specimens, advanced high-strength steels for automotive;
- Non-standard flat specimens, stress triaxiality, and Lode angle;
- Non-standard flat specimens, miniaturization, and size effects;
- Non-standard flat specimens, fatigue;
- Elevated temperature and thermo-mechanical testing;
- Anisotropy and post-necking distortion;
- Cylindrical specimens, standard and non-standard;
- Wire arc additive manufacturing (WAAM).

The next section is dedicated to the Compaction Tension (CT) tests using complex geometry CT specimens, and is centered on the following themes:

- Standard fracture toughness of structural and high-strength steels;
- Extremes like high-temperature and reactor steels, or cryogenic testing;
- Pipeline and pressure vessel steels;
- Welded joints;
- WAAM steel fracture characterization;
- Miniaturized C(T) specimens and novel geometries.

Single Edge Notched Bending, SEN(B), also known as Three-Point Bending (TPB) tests, are used to study material behavior (both steel and concrete) under bending loads that combine compressive, tensile, and shear stress. Therefore, this section is structured around the following research themes:

- Steel characterization, standard fracture toughness and constraint effects;
- Plain concrete, fracture energy and tension softening;
- Plain concrete, size and boundary effects;
- Fiber-reinforced concrete characterization.

Uniaxial compression (UC) tests are the simplest and most effective way of experimental determination of concrete material parameters, spanning the following topics:

- Standard testing procedure and specimen geometry;
- Time-dependent behavior;
- Fiber-reinforced concrete characterization;
- Specialized geometry/interface;
- Sustainable/eco-friendly alternatives;
- Emerging technologies.

The Results and Discussion Section showcases a comparison between reviewed testing procedures, highlighting their advantages, disadvantages, use cases, and state of the art. Finally, the Conclusion summarizes the findings of a comprehensive survey of the recent scientific literature on experimental methods used for testing construction materials and outlines plans for future research.

## 2. Experimental Methods for Construction Materials

### 2.1. Uniaxial Tensile Testing

Uniaxial tensile testing is one of the most common experiments in mechanical engineering, needed to obtain the material’s stress–strain curve. Raw materials are almost always tested by cutting them into dog-bone-shaped samples, even though tension tests can be done on any object (with the use of specialized grips). Dog-bone specimens have a long, narrow shape that widens at both ends to facilitate easier gripping by the testing machine, while the middle section is intentionally narrower to promote failure in the gauge region. Uniaxial tensile testing, as the name suggests, involves subjecting a sample to a uniaxial tension force until it fails. The force applied to the specimen is measured as a function of the displacement between the testing machine’s grips. A typical tension testing machine is vertically oriented, with two grips to hold the material specimen at either end. During the test, the crosshead anchors the top grip, which moves upward. The bottom grip remains static. The crosshead is a beam that can only move up or down; the speed of a tension test is expressed as the “crosshead speed”. Strain gauges and extensometers are the two most important sensors used in tension testing [32]. A strain gauge is a type of load cell device designed to precisely detect forces applied to it and generate an electrical signal of the same magnitude based on the measurement of deformation via resistance change. An extensometer measures length changes. Most extensometers are mechanical devices that are attached to a test specimen prior to the test. As the specimen stretches, the extensometer stretches or extends with it, sending a voltage signal proportional to the degree of extension. However, extensometers can also be virtual, i.e., created using optical metrology techniques, specifically digital image correlation (DIC) [33]. The test sample is painted in a speckle pattern, and the entire test is recorded with one or more cameras. The DIC software (Ncorr v1.2) can compare the subsequent images to the initial “reference” image and calculate the displacement (or strain) across the painted surface [33]. Casita et al. [33]. performed low-cost DIC measurement with a single camera and showed that even with modest equipment, the DIC technique can predict tensile strength and Young’s modulus with good accuracy. More advanced (and expensive) 3D-DIC measurement with two cameras is used by Zhu et al. [34] to monitor the changes of cross-sectional area of the specimen under tension in real time, which is used to obtain true stress–strain curves.

Depending on the material, its application, and test guidelines, dog-bone specimens used in uniaxial tensile testing come in a variety of shapes and sizes. Standards ISO 6892-1 [35] “Metallic materials—Tensile testing—Part 1: Method of test at room temperature” and ASTM E8/E8M [36] “Standard Test Methods for Tension Testing of Metallic Materials” define testing machine specifications, sample geometry, test parameters, and reporting results. E8 uses measuring units used in the USA, while E8M uses metric SI. The key dimensions of every dog-bone specimen are: Lt-total length of specimen, Lc-parallel length, Lo-original gauge length, and So, which is the original cross-sectional area of the parallel length. The gripped ends are connected to the parallel length by a transition (fillet) radius. Original gauge length is the section of the parallel length on which the extensometers are attached.

In this review, the papers in which experiments are done on standard-shaped specimens will be covered, but also, the state-of-the-art papers in which authors had to improvise and think outside of the box to solve a specific problem will be showcased and analyzed.

The choice between cylindrical and flat dog-bone specimens is primarily based on the initial form of the tested material, such as rods or castings in the case of bulk materials, and sheets, plates, or rolled products for sheet metals. Different steel grades have different initial shapes and purposes and are tested according to the intended application and product requirements. Cylindrical specimens are used to test isotropic bulk materials, while the flat specimens are suited for anisotropic sheet materials that undergo work hardening (e.g., rolled steel in the automotive industry).

Mild steel is a low-carbon steel containing ~0.05–0.25% C, characterized by high ductility, toughness, excellent weldability, and machinability. It is supplied mainly as a hot-rolled or cold-rolled sheet/coil, but also as bars, plates, and structural sections. It is used in the automotive industry for body panels and chassis components, but also in building construction for beams, rebar, pipelines, and other general fabrication purposes. For rolled sheets or coils, flat dog-bone specimens are usually cut parallel to the rolling direction to capture anisotropy, while for structural bars or other bulky parts, round specimens are more appropriate.

Cho et al. performed uniaxial tensile testing on the ASTM E8 sub-size standard specimens to study low-carbon mild strength steels that deform inhomogeneously through Lüders band formation [37].

Gonoring, Moreira, and Orlando studied work-hardening of a 0.65 mm thick titanium-stabilized, interstitial-free, cold-rolled, zinc-coated steel sheet, typically used in the automotive industry [38]. Their dog-bone specimens are made according to the ASTM A370 standard, which prescribes mechanical testing of steel products, and covers tensile tests, hardness, bend, impact, and other mechanical property tests [39]. For uniaxial tensile testing of sheet steels, A370 specifies narrower ranges of flat specimen dimensions, as it focuses solely on the analysis of steel products.

Van der Heijde and Samad studied the effect of specimen thickness on the Lüders phenomenon in AISI 1524 hot-rolled steel alloy using flat, 1, 2, 3, and 4 mm thick dog-bone specimens [40] according to the ASTM E8/E8M standard [36]. They concluded that Lüders band width and propagation velocity increase with thickness, while Lüders strain decreases.

Parapurath et al. analyzed the effect of heat treatments on the microstructure and the corrosion behavior of S275 mild steel in different chemical and acidic environments [41]. They prepared flat dog-bone specimens according to ASTM A370 and ASTM E8 standards. Quenching and tempering were performed in two different heat-treatment cycles. The mechanical behavior of the material was analyzed for the original, quenched, and tempered material, with every test repeated five times for accuracy and consistency. Parapurath et al. showed that quenched martensitic microstructures significantly increased hardness and strength, while reducing corrosion rates compared to original and tempered states [41].

Rebeyka et al. investigated the mechanical behavior of HSLA350/440 and DP350/600 steel grades in order to calibrate the Hensel–Spittel constitutive equation [42]. High-strength low-alloy (HSLA) and dual-phase (DP) steels are both used in the automotive industry. HSLA steels consist of a ferrite matrix with fine carbides and nitrides, while DP steel is characterized by a microstructure of soft ferrite matrix and hard martensite islands, giving it a unique combination of high strength and good ductility. Rebeyka et al. [42] tested them according to the ASTM E8 standard [36] and ASTM E21 [43] “Standard Test Methods for Elevated Temperature Tension Tests of Metallic Materials”. The testing was done in the temperature range from 30 °C to 800 °C and at strain rates from 0.035 s^−1^ to 1.35 s^−1^, simulating conditions for the motor vehicle parts manufacturing process.

Zhang et al. studied the size effect on the post-necking behavior of dual-phase 800 steel in order to determine the feasibility of using a miniaturized non-standard tensile specimen to test rapid alloy prototyping [44]. They compared the standard ISO and ASTM size specimens with the miniaturized ones and determined that the proposed mini specimens can accurately measure tensile strength and elongation but fail to capture true post-necking behavior, like fracture angle, necking mode, and triaxiality, if the aspect ratio of the tensile specimen’s parallel width to its parallel thickness is less than 5.

Zheng et al. also studied miniaturized tensile specimens [45]. They performed a rigorous review of specimen size, geometry, preparation, measurement technique, and data analysis.

Hwang studied miniature specimens that adhere to ASTM guidance concerning the gage length to diameter ratio of 5 but have a diameter less than the minimum ASTM size [46]. His research focus was on twinning-induced plasticity of Fe-17.3Mn-0.64C-1.38Al steel, more specifically, on specimen preparation effects. He concluded that lathe machining introduced surface deformation twins, which increases yield strength and reduces elongation in smaller diameters, so Hwang recommends using specimens with a gage diameter >5 mm or using polished specimens that show size-independent properties.

Alar and Mandić used uniaxial tensile testing according to the ISO 6892-1 standard [35] to verify their claim that the Charpy impact testing method can be used for evaluating the mechanical properties, such as yield strength and tensile strength [47]. For high-strength steel designated as API 5L X80, using Charpy impact tests, they obtained results that differed by less than 5% from those measured in tensile tests.

Larour et al. studied edge crack sensitivity in advanced high-strength steels (AHSSs) [48]. They used 300 samples of cold-rolled sheet steels with a tensile strength range between 600–1200 MPa and proved that conventional tensile properties, such as yield strength, tensile strength, elongation, etc., correlate poorly with the hole expansion ratio (HER) results obtained according to the ISO 16630 standard [49] “Metallic materials—Sheet and strip—Hole expanding test”.

Çavuşoğlu, Güral, and Gürün studied the influence of strain rate on tensile properties and fracture behavior of DP600 and DP780 dual-phase steels [50]. They used standard ASTM E8 uniaxial tensile specimens [36] cut in both rolling (RD) and transverse (TD) directions, performing tests at quasi-static strain rates (0.001, 0.01, 0.06 s^−1^). They concluded that increasing the strain rate leads to an increase in yield strength, tensile strength, elongation, and hardening rate of DP600 and DP780 dual-phase steels, while the strain hardening coefficient remains unaffected by the change.

In their highly cited paper [51], Heibel et al. performed uniaxial testing of dual-phase (DP600, DP800, DP1000) and complex-phase (CP800, CP1000) sheet steel for automotive applications, according to the ISO 6892-1 standard [35], and developed a new classification scheme for high-strength multiphase steels based on global and local formability.

Świłło and Cacko studied DC04, a cold-rolled, non-alloy steel with a low carbon content, which is known for its excellent deep-drawing properties, high surface quality, good weldability, and exceptional formability [52]. Due to the purpose of this steel, a post-necking true stress–strain curve is required, so the authors developed a low-cost, computationally efficient, vision extensometer with image processing (VEIP) as an alternative to DIC. Świłło and Cacko carried out their experiments according to outdated national (Polish) standards, which prescribe the same specimen geometry as the ISO 6892-1 standard [35] but do not include non-contact extensometry.

Mirone et al. studied anisotropy and progressive cross-section distortion in the post-necking regime of anisotropic thin sheet metals, namely mild steels CR04 and HR12, as well as high-strength steels MTC1300T and FORTIFORM, using only a single-camera setup to avoid complex 3D DIC systems [53]. They employed non-standard flat dog-bone specimens with a width/thickness ratio < 3 in order to mitigate the distortion of the necking section.

Steel is often formed at elevated temperatures, and to conduct a realistic study of its properties under working conditions, specialized equipment is required. Zhang et al. studied zinc-coated boron steel AHSS 22MnB5 using a Gleeble 3800 (Dynamic Systems Inc., Poestenkill, NY, USA) thermal–mechanical physical simulation system, a research instrument designed to replicate industrial thermal and mechanical conditions [54]. Generally, material testing at elevated temperatures is prescribed by the standards ISO 6892-2 [55] and ASTM E21 [43]. Zhang et al. [54], however, used standard, half-length, and half-width specimens to show that these standards do not account for the non-uniformity of the temperature distribution along the gauge length and proposed the development of new standards specifically tailored for thermo-mechanical testing with thermal gradients.

A recurring motivation across multiple test types is the deliberate manipulation of the local stress state. Under standard uniaxial loading, a uniform gauge section produces near-zero stress triaxiality and a fixed Lode angle. However, calibration of advanced multiaxial damage evolution models, such as the Johnson–Cook or Bai–Wierzbicki criteria, requires experimental data spanning a range of stress triaxiality and Lode angle combinations. This is the fundamental mechanical rationale for the non-standard specimen geometries (notched, grooved, shear, central-hole) that appear throughout this section: each geometry is engineered to shift the local stress state in a controlled and predictable way, expanding the calibration space of the damage model beyond what a single standard uniaxial test can provide. The present review documents these geometries and their procedural context; the numerical implementation of the resulting parameters lies outside its scope.

**Figure 2:**
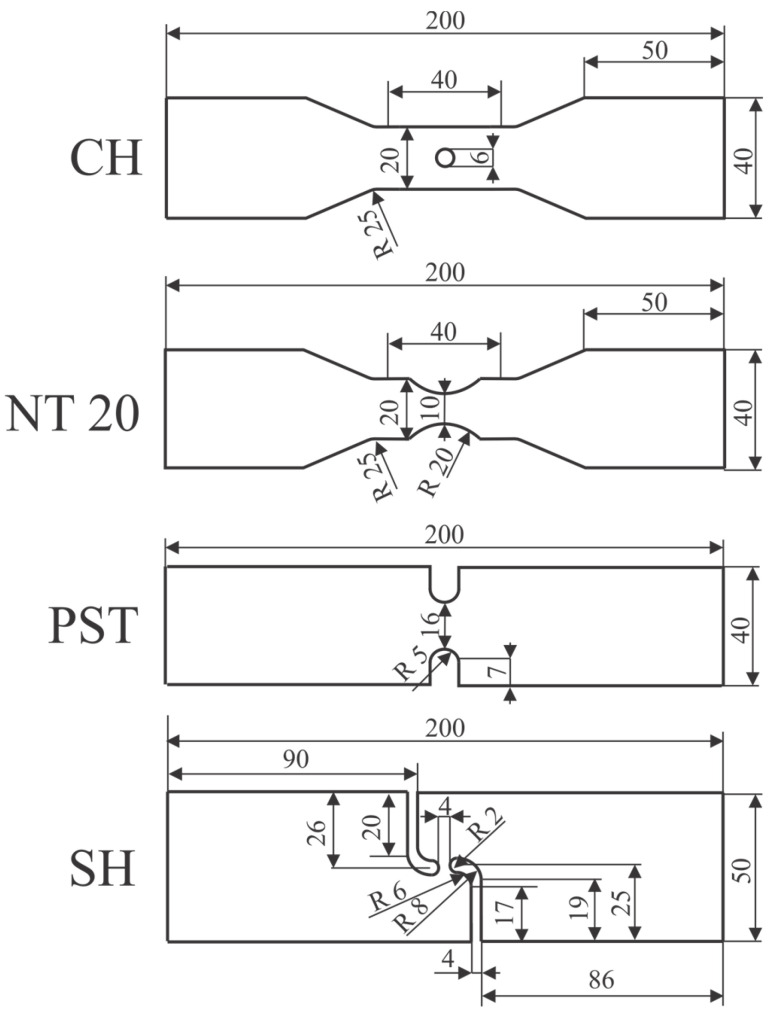
Specimen geometry used by Cerik and Choung [56].

Cerik and Choung studied ductile fracture in three shipbuilding steels: mild A-grade (KR), high-strength AH36 (ASTM A131), and DH36 (LR) [56]. Their prediction model combines normalized Cockcroft–Latham and maximum shear stress criteria, accounting for stress triaxiality and Lode angle effects. They used standard flat dog-bone specimens, but also four research-specific geometries designed to address different stress states (combinations of stress triaxiality and Lode angle). Notched tension specimens (NT20) have a large notch radius with a narrowed gauge section, which produces a relatively well-defined fracture initiation point. Plane-strain tension (PST) specimens generate higher constraint and non-proportional loading paths after necking. NT20 and PST specimens are tailored to study fracture under moderate-to-high triaxiality. Central-hole specimens (CH) create a biaxial stress state with lower triaxiality than notched bars. Shear specimens (SH) used by Cerik and Choung are a variation of Peirs geometry [57], designed to have low triaxiality and shear-dominated stress states for capturing the role of the Lode angle parameter. The geometry of specimens used by Cerik and Choung is shown in Figure 2.

Gopinath et al. also used a custom geometry of dog-bone specimens in order to determine Johnson–Cook constitutive and damage parameters for E250 structural steel [58]. They used a combination of regular, notched, and grooved specimens to achieve different stress triaxiality.

Another example of pure shear geometry can be found in the work of Wang et al. [59]. They also used cylindrical dog-bone specimens, notched cylindrical dog-bone specimens, flat grooved dog-bone specimens, cylinders, and notched cylinders made from Q550, Q690 and Q890 grade HSS. They examined the applicability of the von Mises yield criterion and concluded that the influence of stress triaxiality is negligible, while the effects of the Lode angle are significant in HSS, especially in shear and compressive conditions.

Shen et al. studied local formability in advanced high-strength, medium-Mn steels using standard smooth dog-bone specimens for tensile properties and flat notched geometries (shear, central hole, plane strain) to capture stress-state effects [60]. They showed that medium-Mn steels have superior global ductility and hardening but inferior local formability in comparison to the DP1000 high-strength steel grade.

Similarly, Pan et al. used standard dog-bone, notched, central-hole, and plane-strain samples cut along three orientations, 0°, 45°, and 90° relative to the rolling direction, in their study on failure and formability in dual-phase DP780 steel [61]. They used the Bai–Wierzbicki damage model with the Swift–Voce hardening law in order to predict early fracture before necking.

Another similar methodology was presented by Xin and Veljkovic, who studied the fracture material properties based only on the standardized uniaxial stress–strain curve [62]. For this, they combined a rate-independent nonlinear isotropic J2 hardening model with a separate Hosford–Coulomb fracture model using ABAQUS (v 6.14) user subroutine VUMAT. Their specimens were made of S700 and S960 HSS plate with standard geometry, as well as CH with different hole sizes (8, 16, 24, 32, and 40 mm), with the purpose of stress concentration effects analysis. They used FEM simulation of the K gap joint to demonstrate that the proposed fracture model can be applied to predict fractures in real structures.

Azinpour et al. studied the application of the plastic work threshold value in phase-field numerical procedures and its influence on inelastic and post-critical material behavior [63]. For result validation, they used a flat grooved tensile specimen and a notched plate tensile specimen similar to NT20 and PST used by Cerik and Choung [56]. For the flat grooved specimen test, they used a sample made from an aluminum alloy with a notch radius of 7 mm and applied a displacement-driven boundary condition. They used these experimental results for comparison with the simulations based on the residual control staggered algorithm (RCSA) and the standard staggered algorithm (SSA). For the notched plate tensile test, they used a miniature-sized tensile sheet specimen made of austenitic stainless steel 316L. They compared the force-displacement diagrams from the experiments with the simulations using different plastic threshold values and performed an evaluation of the numerical performance of the specified phase-field model. This paper showcases the versatility of the phase-field model and its application to stainless steel as well as an aluminum alloy, and it features theoretical consideration, numerical simulations, and finally, experimental verification.

The study done by Kriaa et al. [64] focuses on the prediction of brittle fracture using the phase-field approach. The authors conducted experimental tensile tests on non-standard double-notched plates made of quenched non-alloy steel C90 to validate the phase-field model. The specimens had varying notch axial distances (h = 5 mm and 10 mm) to induce different crack trajectories and were subjected to quasi-static tension, so the crack patterns in the specimens were compared with the numerical results obtained using the hybrid formulation of the phase-field model.

Zhang et al. studied the post-necking work hardening behavior of S690QL steel using a standard uniaxial tensile machine [65]. They used standard flat 10 mm thick dog-bone specimens cut at 0°, 45°, and 90° angles relative to the rolling direction to obtain the pre-necking strain hardening behavior. They developed an inverse identification procedure to determine the post-necking strain hardening behavior, which uses the Finite Element Model Updating (FEMU) technique combined with DIC. They showed that plastic anisotropy affects the identification of the post-necking strain hardening behavior. To account for this, they developed a strategy to extract the pre-necking 3D plastic anisotropy using a custom geometry double-notched tensile specimen.

Kreithner, Niederwanger and Lang studied the influence of the ductility exponent on the fatigue of structural steels, namely S355 and S700 [66]. They first performed uniaxial testing on flat tensile specimens according to ISO 6892-1 [35] to obtain stress–strain curves for those two steels and concluded that the mild structural steel S355 has greater ductility, shown by the elongation at break of 30.97%, while the high-strength structural steel S700 has elongation at fracture of 20.05%. To study the fatigue performance of these steels, they performed cyclic testing in the force-controlled setup using custom flat specimens with a drilled-in hole, which introduces stress concentration and controlled crack initiation, which deviates from the regulations prescribed by standards ISO 12106 [67] “Metallic materials—Fatigue testing—Axial strain-controlled method”, ISO 1099 [68] “Metallic materials—Fatigue testing—Axial force-controlled method”, ASTM E606/E606M [69] “Standard Test Method for Strain-Controlled Fatigue Testing”, and ASTM E466 [70] “Standard Practice for Conducting Force-Controlled Constant Amplitude Axial Fatigue Tests of Metallic Materials”. ISO 12106 [67] and ASTM E606/E606M [69] standards allow both notched and unnotched specimens, but they also require strain-controlled cycles, while ISO 1099 [68] and ASTM E466 [70] prescribe force-controlled testing but require smooth, unnotched specimens.

Another example of fatigue testing using flat dog-bone specimens can be found in the paper by Zvirko et al. [71]. They studied the mechanical behavior of low-carbon rolled steel after a 33-year operation of a portal crane. They concluded that different parts of the crane are subjected to different load intensities and subsequently have different mechanical properties as a result of strain hardening. They made two sets of test specimens, one for high and the other for low cyclic stress ranges, and concluded that non-uniform (post-necking) elongation is the most sensitive tensile parameter for assessing operational degradation in rolled steels.

Huang et al. studied the mechanical properties and microstructural composition of wire arc additively manufactured (WAAM) steels [72]. They tested 137 flat dog-bone specimens according to ISO 6892-1 [35], with some specimens being rough and in as-built condition, while the others were machined to achieve a smooth surface. Two grades of material were tested, ER70S-6 and ER110S-G, with two plate thicknesses of 3 mm and 8 mm, which were cut into specimens at 0°, 30°, 45°, 60°, and 90° relative to the print layer direction to probe anisotropy. Their research showed that WAAM steels exhibit slightly reduced strength due to slower cooling. They also compared multiple WAAM deposition strategies and concluded that there is no significant difference in tensile properties for the tested methodologies, except for some variation in ductility.

Moving on to the testing of cylindrical dog-bone specimens, they are machined from round bars and have excellent surface finish, which provides ideal uniaxial conditions in the gauge section: uniform stress distribution under axial load and reduced edge effects, giving more accurate stress–strain data, which is important for material constitutive modeling or calibration. They are easier to align perfectly in a testing machine, but a round surface makes DIC very impractical.

Kassaye et al. made a direct comparison between flat and cylindrical dog-bone specimens made from the same material, 0.17C-5Mn-0.76Al-0.9Si-Nb medium manganese quenching and partitioning (QP) steel [73]. Flat specimens were machined from 7 mm thick plates, while the cylindrical ones were made from 12 mm thick plates. The experiments performed by Kassaye et al. [73] provided similar measured tensile strengths of 1400 MPa for 12 mm cylindrical specimens and 1325 MPa for 7 mm flat specimens, while the total elongations had a greater difference: 15% for cylindrical and 11% for flat specimens.

Harwell, Spears, and Ebrahimpour developed failure-limit material models for Aluminum 6061-T6, A36 Carbon Steel, 304 Stainless Steel, and Nitronic 60 Stainless Steel, based on parameters of plastic equivalent strain (failure strain) and stress triaxiality [74]. In order to obtain all the necessary data required for the development of such a comprehensive material model, they used a total of 11 different specimen geometries: cylinder, smooth round bar, large-notched round bar, small-notched round bar, solid square bar, flat grooved plate, simple shear plate, combined shear/tension plate, perforated plate, pipe, and cylindrical dog-bone. Many of the custom, non-standard specimen shapes used by Harwell and coauthors are inspired by geometries used by Bao and Wierzbicki to study fracture criteria and stress states in aluminum alloy [75].

Paul et al. developed a simplified procedure to determine the post-necking true stress–strain curve from the uniaxial tensile test performed on the cylindrical specimens with a 7 mm gauge diameter and made of rail steel [76]. For the calculation of the Bridgman correction factor, the a/R ratio is needed, but experimental measurement of a and R is tedious and time-consuming, so Paul et al. [76] proposed a new correction factor requiring only uniform elongation and local axial strain and avoiding complex neck profile measurements.

Yao and Wang studied the strain hardening behavior of structural steels beyond the necking occurrence [77]. They used standard cylindrical specimens made of ordinary structural steels Q235 and Q355, high-strength steel Q460, and low-yield-point steel LYP225. They developed an image-processing method that can extract the necking profile of cylindrical dog-bone specimens from the 2D grayscale images and can be used to measure the instantaneous minimum radius and necking curvature required by Bridgman’s theory to calculate equivalent von Mises stress.

Sey and Farhat investigated hydrogen embrittlement in AISI 1018 mild steel using sub-size cylindrical tensile specimens [78]. These sub-size specimens with 3.3 mm diameter have a higher surface-to-volume ratio in comparison to the full-size specimens, so the hydrogen introduced electrochemically diffuses more quickly and uniformly through the smaller cross-section.

Similarly, Madi et al. studied hydrogen embrittlement in E355 pipeline steel using miniaturized tensile specimens with diameters of 1.2 and 2.4 mm, which are placed in a pressure chamber incorporated into an MTS hydraulic machine [79]. This chamber can withstand pressures up to 250 bars, making the experiments performed by Madi et al. [79] more realistic and closer to the real working conditions for which E355 is designed, but it also posed a challenge in terms of strain measurements. The used pressure chamber has four sapphire windows, which enable the utilization of the Edge Tracing technique for optical extensometry.

Matsuno et al., on the other hand, studied steel tubes used in the automotive industry using miniaturized tensile specimens (1.0 mm gauge diameter) [80]. They studied anisotropic behavior in the axial and hoop directions caused by the cold drawing. As the analyzed steel tubes had a diameter of 40.6 mm and a thickness of 1.8 mm, the axial specimens could be machined out directly from the tube, while for the hoop specimens, Matsuno et al. [80] had to laminate a dummy tube onto the test tube using electron beam welding in order to have enough material to properly cut out the gripped ends. They analyzed three tubes made from SAE1017, E275, and a proprietary hyper-burring automotive steel grade with bainitic ferrite microstructure. They observed that steel tubes can have inferior, the same, and superior fracture strain in the hoop direction in comparison to the axial direction, which can deteriorate by over 40% in the worst case due to the combination of grain elongation and carbide aggregation.

Dzioba et al. used standard cylindrical specimens with diameters of 5 and 10 mm, made of S355 steel, to study the constitutive relationship during the loading conditions where plastic deformation is significant, i.e., in front of a crack tip or in a neck [81]. Their innovative approach is based on combining several measurement techniques, including video neck tracking, electrical potential monitoring, and grain-scale strain analysis. This multi-modal approach enabled calibration of constitutive stress–strain relations beyond uniform elongation and was validated with FEM simulations.

Mroziński et al. studied the effect of microstructural anisotropy on the low-cycle fatigue properties of S420M steel [82]. Their cylindrical dog-bone specimens were made both perpendicular and parallel to the rolling direction of 40 mm thick sheet metal. Their tests included static tensile test, hardness, microstructural analysis, and low-cycle fatigue under constant-amplitude and programmed loading, and were performed according to EN 10164 “Steel products with improved deformation properties perpendicular to the surface of the product—Technical delivery conditions” [83]. They demonstrated that the static strength does not depend on the orientation, while fatigue life is drastically reduced (between 50–300%) in perpendicular specimens.

Wang et al. [59] analyzed the yield criterion for HSS, which has higher strength but lower ductility than mild steel and is therefore more brittle. Consequently, the classical von Mises yield function fails to capture the plastic behavior of HSS under complex stress states due to the neglected influence of stress triaxiality and the Lode angle [59].

The most out-of-the-ordinary paper about uniaxial tensile testing that will be covered in this review is published by Donnini et al., who used dog-bone shaped specimens made from ultra-high performance fiber-reinforced concrete (UHPFRC), tested on a machine with a load capacity of 50 kN and displacement control, with a loading rate of 0.5 mm/min and a custom-built gripping fixture [84]. The matrix was mixed using Portland–limestone blended cement CEM I 52.5 R, quartz micro-sand with particle sizes of 0–0.6 mm and 0.6–1.0 mm, silica fume, and acrylic-based water-reducing admixture (WRA). The water-to-cement ratio (w/c) was 0.20. Donnini et al. [84] reinforced this matrix with hooked steel fibers and studied the effects of different fiber amounts (0%, 0.6%, 1.25%, 1.9%, and 2.55% volume fraction) on the mechanical behavior and post-elastic evolution. The total length of dog-bone specimens was Lt = 330 mm, with a_0_ = 30 mm and b_0_ = 45 mm in the cross-section. Deformation was measured using three digital cameras to obtain stereo-3D-DIC measurements, and the experiment was simulated using a phase-field model that has been implemented in an FEM code. Donnini et al. [84] concluded that the inclusion of hooked steel fibers greatly improves the uniaxial tensile behavior of UHPFRC specimens, even at a low fiber fraction of 0.6%, and they studied two main failure processes: quasi-brittle and ductile.

### 2.2. Compact Tension Tests C(T)

These tests are specifically designed to study fracture mechanics and crack propagation behavior in structural steels and other brittle and quasi-brittle construction materials [85]. C(T) specimens are more economical in terms of the number of pieces that can be created from sampled thick plates but are more expensive in terms of machining costs. Unlike uniaxial tensile tests, which provide global strength and ductility parameters, C(T) tests are used to predict local mechanical response if the construction has a crack. The specimens used in these experiments have complex geometry designed to obtain a sharp and reproducible crack tip and require a lot of machining operations. For rolled materials, the notch should be aligned with the rolling direction, as this is the direction in which the material is weakest. This ensures that all results are on the safe side.

The material’s fracture toughness and its behavior under stable crack growth are studied according to standards ASTM E399 [86] “Standard Test Method for Linear-Elastic Plane-Strain Fracture Toughness K_Ic_ of Metallic Materials”, ASTM E647 [87] “Standard Test Method for Measurement of Fatigue Crack Growth Rates” and ASTM E1820 [88] “Standard Test Method for Measurement of Fracture Toughness”, which prescribes testing needed to obtain elastic–plastic parameters such as the J-integral, stable tearing resistance (J–R curves), and Crack Opening Displacement (COD) like Crack Tip Opening Displacement (CTOD), or Crack Mouth Opening Displacement (CMOD). Other important fracture properties that can be obtained using C(T) tests are stress intensity factor (SIF), usually marked as K_Ic_, and critical fracture energy G_Ic_. ASTM E2472 [89] “Standard Test Method for Determination of Resistance to Stable Crack Extension under Low-Constraint Conditions” prescribes C(T) testing for measurement of the Crack Tip Opening Angle (CTOA), a material parameter used to predict ductile tearing in pipelines. ASTM E1921 [90] “Standard Test Method for Determination of Reference Temperature, To for Ferritic Steels in the Transition Range” introduces elastic–plastic equivalent stress intensity factor K_Jc_, which is derived from the J-integral at the onset of cleavage fracture (J_c_). K_Jc_ is used to construct the master curve and determine its fitting parameter: reference temperature T_0_.

ISO alternatives to these ASTM standards are less referenced but still worth mentioning: ISO 12737 [91] “Metallic materials—Determination of plane-strain fracture toughness”, and ISO 12135 [92] “Metallic materials—Unified method of test for the determination of quasistatic fracture toughness”.

The simple geometry defined by ASTM standards is the most practical and cost-effective solution for specimen design if the digital image correlation (DIC) is used for COD measurement. However, if the COD is measured using the Double-Cantilever Clip-In Displacement Gage, the specimen should also feature machined Integral Knife Edges.

Suetrong and Uthaisangsuk tested fatigue crack propagation in ER8 high-speed railway wheel steel [93]. They used sub-size tensile specimens prepared according to the ASTM E8 standard [36] and C(T) specimens made according to the ASTM E647 standard [87]. Afterwards, they compared the experimental results with the numerical simulations performed using the extended FE method (XFEM) and obtained material constants C and m for the Paris equation.

İriç et al. studied limitations of C(T) specimens, in particular, the minimum thickness required for valid plane-strain fracture toughness determination using medium-strength AISI 1040 carbon steel [94]. They investigated the effect of the heat treatment process on the minimum thickness calculation, so they compared untreated specimens with the heat-treated (quenched and tempered) and notched after heat-treating specimens, and concluded that heat treatment significantly increased yield strength and fracture toughness while reducing the minimum thickness, with the notched after heat-treating specimens having the smallest required thickness.

Newman performed a study on fatigue and crack growth in a low-carbon, nickel–chromium–molybdenum carburizing steel alloy 9310, which has a wear-resistant surface with a ductile core and is commonly used in the aerospace industry for gears, shafts, and bearings [95]. He used compression pre-cracking instead of load-shedding to improve near-threshold reliability and developed the “Rainflow-on-the-Fly” algorithm implemented in FASTRAN life-prediction code [96], which represents a unified fatigue approach, from crack nucleation (small-crack growth) and large-crack growth to failure.

Thamaraiselvi and Vishnuvardhan studied pressurized thermal shock load, which is one of the major factors affecting the lifespan of a reactor pressure vessel [97]. They used standard C(T) specimens made from 20MnMoNi55 steel used in reactors and performed a series of experiments—tension at room temperature, tension at 300 °C, and thermal shock with tension—and concluded that the fracture toughness is sensitive to temperature, reduction in fracture toughness after thermal shock is caused by the increase in brittleness, the slope of the J-R curve decreases with temperature, and the percentage reduction in J-integral observed after thermal shock experiments is similar to that of irradiated specimens found in the literature.

Park et al. analyzed Charpy impact energy and J-integral fracture toughness values of high-Mn steel and 304L stainless steel at room and cryogenic temperatures down to −196 °C [98]. They used C(T) specimens with side grooves on both sides, made in accordance with ASTM E1820 [88], as well as Charpy V-notch specimens, and concluded that J-integral fracture toughness provides a more reliable measure of cryogenic fracture resistance than Charpy impact energy.

Janulionis et al. studied the fracture toughness of aged high-strength ferritic–martensitic steel P91, which is designed for extreme high-temperature service, mostly in power generation and petrochemical industries [99]. It has high thermal stability and stress rupture resistance due to excellent creep strength at elevated temperatures, but because of its intended purpose, it is difficult to study experimentally, as it would require specific, realistic working conditions, so Janulionis et al. [99] used FEM analysis backed up by experimental results for their methodology of numerical determination of fracture toughness. In this paper, uniaxial tensile testing was performed using cylindrical specimens, according to the ISO 6892-1 [35] and ISO 6892-2 [55] standards for room and elevated temperatures, respectively, while the fracture toughness experiments were carried out according to ASTM E399 [86].

Similarly, Bajić used simulated aging (specimens heated at 250 °C for 30 min) to obtain specimens with equivalent properties to those of a 50-year-old hydropower plant pipe made from NIOVAL47 steel [100]. He used cylindrical dog-bone specimens and C(T) specimens in experiments conducted according to the ASTM E1820standard [88]. In accordance with the SINTAP procedure [101] and using FEM software KOMIPS [102], he determined that the critical crack depth is reached before “leak before break” condition in many areas with high stress concentration, so catastrophic failure would occur; therefore, it is necessary to introduce an online monitoring system.

Beltrán-Zúñiga et al. developed a new methodology for the determination of the fracture toughness K_Ic_ for low-carbon steel pipes using full-sized C(T) specimens created by welding attachments to the pipe cross-section [103] to meet ASTM E399 and ASTM E1820 requirements. Due to the production process and composition, the analyzed API 5L X46 steel has anisotropic properties in the longitudinal (L), circumferential (C), and radial transverse (R) directions. Their research showed that the K_Ic_ values in the short transversal direction of low-carbon steel pipes are up to 20% lower than the values in the CL direction.

Ermakova et al. investigated fatigue crack growth behavior in wire arc additively manufactured (WAAM) ER70S-6 low-carbon steel [104]. The C(T) specimens are extracted in vertical and horizontal orientations and studied according to ASTM E647 [87] and ASTM E1820 [88] standards under cyclic loading of 10 kN and 11 kN. Ermakova et al. [104] showed that fatigue life differs with orientation and is also heavily dependent on load, so when they increased the load from 10 to 11 kN, the test duration decreased by approximately 2.3 times for horizontal specimens and 1.5 times for vertical specimens.

O’Neill et al. studied fatigue cracking and the redistribution of residual stresses in steel produced by the WAAM technique using ER100S-1 welding wire [105]. They used Electro Discharge Machining (EDM) to create two Compact Tension C(T) specimens made according to the ASTM E647 standard [87], while the clip gauge knife edges were machined on the specimens according to the ASTM E1820standard [88]. O’Neill et al. [105] combined surface treatments (laser shock peening and rolling) with overload fatigue cycles and performed advanced residual stress characterization via neutron diffraction. They also performed FEM analysis to test the hypothesis that the fatigue cracking procedure at high load would result in a residual stress pattern similar to overloading.

Sales et al.’s research focused on super duplex stainless steels (SDSSs) fabricated using the WAAM technique and fatigue crack growth rates, with the net effect of crack tip shielding mechanisms [106]. Their C(T) specimens were made so that cracks propagate longitudinally and transversely to the WAAM deposition direction, and the results showed that the fatigue life is anisotropic, with specimens with the notch parallel to the deposition direction lasting ~50% longer than specimens with the notch cut normal to the deposition direction.

Petkov et al.’s research focus was on creep crack growth analysis within the heterogeneous weld structures [107]. They used Cross-Weld specimens, which have non-standard dimensions and which were tested using uniaxial testing, as well as C(T) specimens. Their specimens include parent material (PM), which is also often called base material (BM), weld metal (WM), and heat-affected zone (HAZ), but ISO 6892-1 [35] and ISO 6892-2 [55], as well as the ASTM E8/E8M standard [36], require that specimens be made from homogeneous material to have a uniform stress state, so the work by Petkov et al. [107] is a research-motivated adaptation to study the structural behavior of the welded joint. These CW specimens can be symmetrical (HAZ centered) and perpendicular (HAZ aligned perpendicular to load direction). Similarly, C(T) specimens can have a weld-centered crack, a crack on the interface, and a crack asymmetrically positioned with regard to the welded joint. Petkov et al. [107] analyzed C(T) specimen stress field orientation and complications arising from simulations with different material models.

Similarly, Song et al. investigated fatigue crack growth in 2.25Cr1Mo0.25V steel welded joints using C(T) specimens made from BM, WM and HAZ [108]. They used acoustic emission to successfully monitor crack evolution. Their microstructural analysis linked coarse grains and inclusions to higher crack growth rates, proving that BM has superior fatigue resistance, HAZ has the poorest at low stress intensity, and WM is undermined by residual stress, but the formation of numerous secondary cracks (linked to coarse grains and inclusions) consumes energy during the crack propagation, which eventually lowers the fatigue crack growth rate in WM.

Jiang et al. studied the effect of heat input during welding on the fracture toughness of five HSS grades (marked as QT550, QT690, QT890, TMCP550, TMCP690), which have a yield strength of 550 MPa, 690 MPa, or 890 MPa, with two delivery conditions: the first being quenched and tempered (QT), and the second being thermo-mechanical control process (TMCP) [109]. They used standard C(T) specimens made according to ASTM E1820 [88] and performed a comparative study with pre-crack located in BM, HAZ, and WM. Based on the experimental findings, they determined the optimal heat input for quenched and tempered HSS welding to achieve satisfactory fracture toughness. Jiang et al. [109] concluded that an increase in steel strength leads to a decrease in the optimal heat input for QT HSS, and that an increase in yield strength results in an increase in fracture resistance for both QT and TMCP HSS.

Liu et al. used acoustic emission to monitor crack evolution and to distinguish damage sources: cyclic plastic deformation, tensile crack fractures, and shear crack fractures [110]. Using the CO_2_ gas arc welding technique and ER50-6 weld wire, they created a 20 mm thick welding plate consisting of G20Mn5QT and Q345D steel grades. C(T) specimens were machined using Wire Electrical Discharge Machining (WEDM) according to the ASTM E647 standard [87]: one entirely made from G20Mn5QT, and the other with the crack along the middle of the welded joint. The tests were carried out under sinusoidal cyclic loads with a maximum load of 18 kN and a load ratio of 0.5. Two narrow-band piezoelectric ceramic transducers were used to record the signals, which were sorted by the K-means cluster algorithm.

Song et al. studied fatigue crack growth in D32 marine structural steel overmatched welded joints using JQ501-1 as filler metal [111]. Overmatching welding implies using filler material with greater strength than base material, and it is common in marine, offshore, and pressure vessel applications because it moves stress and strain concentration from the WM into BM or HAZ. Song et al. [111] fabricated C(T) specimens using the electron discharge machining (EDM) technique, and testing was performed according to the ASTM E647 standard [87]. The results showed that residual stress in the overmatched welded joints leads to a decrease in fatigue crack growth rates, and that the NASGRO equation can be used for their modeling across different zones and stress ratios.

Ma et al. studied hydrogen embrittlement that occurs in hydrogen storage vessels, with the assumption that cracks have already begun to form and are present at the pressure vessel’s high-stress locations [112]. The first cracks spread under fatigue load until the crack depth reaches the critical value, which is established in accordance with the guidelines that state that the vessel failure should occur as a leak-before-burst mode and that the maximum SIF at the crack tip stays less than the subcritical cracking threshold (K_IH_) of 45 MPa. Ma et al. [112] machined side grooves along the specimens’ side faces in the same plane as the pre-crack starter notch. Every specimen was taken from the actual vessel’s cylinder, made from Cr–Mo 4130X, a high-strength low-alloy (HSLA) steel that is quenched and tempered and optimized for high-pressure vessels but vulnerable to hydrogen-assisted cracking, and the direction of sampling ensured that the loading and crack-growth directions ran parallel to the vessel’s longitudinal and circumferential axes, respectively.

Sandia National Laboratories, in collaboration with the US National Science Foundation and the Naval Surface Warfare Center Carderock Division, announced a computational challenge presented in the paper by Boyce et al. [113]. The goal of the challenge was to test the predictive capabilities of state-of-the-art numerical implementations, computational techniques, and physics models. Scientists and engineers were asked to forecast the beginning and spread of cracks in a straightforward but unique geometry made from 15-5 PH precipitation-hardened martensitic stainless steel, heat-treated to the H1100 condition, which is a typical commercial engineering alloy that is readily available off the shelf. Specimens included dog-bone, C(T), and custom fracture challenge geometries with blunt notches and interacting holes. Standard C(T) specimens were used to provide fracture toughness/tearing calibration data, while the novel, custom fracture challenge geometries had geometries similar to C(T), leaving researchers without prior knowledge of experimental outcomes and ensuring unbiased fracture prediction.

Tobita et al. studied the application of miniaturized 0.16T-C(T) specimens made from reactor pressure vessel steels [114] for fracture toughness evaluation using the master curve method defined in the ASTM E1921 standard [90]. They tested five SA533B Cl.1 steels (JRH, JRM, Steel A, Steel B, JRL) with varying ductile-to-brittle transition temperatures. Their results showed that *T*_0_ calculated from the results of miniaturized 0.16T-C(T) specimens has good agreement with *T*_0_ calculated using full-size 1T-C(T) specimens, and that the loading rate effects were consistent across sizes.

Similarly, Sanchez et al. also used mini-C(T) specimens (4 mm thick) for master curve analysis [115], according to the ASTM E1921 standard [90], but they analyzed four structural steels—S275JR, S355J2, S460M, and S690Q—and showed that mini C(T) specimens accurately model their fracture behavior, with the calculated reference temperature *T*_0_ being within ±30 °C, compared to results from full-size specimens.

Another example of non-standard geometry can be found in a paper by Di et al., who studied CTOA, a fracture parameter used in ASTM E2472 [89] to characterize failure in high-pressure pipelines [116]. Their modified compact tension (MCT) specimens were cut directly from X80 steel pipes in the circumferential–longitudinal direction and machined into flat specimens, with the crack plane oriented in the circumferential direction, so that crack propagation occurs in the longitudinal direction, which, along with a wider and longer tearing ligament, provides steady-state crack propagation.

### 2.3. Single Edge Notched Bending Tests: SEN(B)

Single Edge Notched Bending (SEN(B)) tests, often denoted as (SENB) or referred to as three-point bending (TPB), are used to test both metals and concrete. They characterize the crack initiation and crack growth resistance of materials under bending-dominated loading. For metals like steel, they are defined in standards ASTM E399 [86], ASTM E1820 [88], and ISO 12135 [92], which also define C(T) specimens’ geometry and properties, as shown in the previous chapter. The standard that is specific to SEN(B) testing is ISO 15653 [117] “Metallic materials—Method of test for the determination of quasistatic fracture toughness of welds”.

Unlike metals, concrete fracture is nonlinear and quasi-brittle, so classical Fracture Toughness K_Ic_ or J-integral at the onset of cleavage fracture (J_c_) are not appropriate. Instead, in the case of concrete, fracture energy G_F_, effective fracture toughness, load–CMOD and load–deflection curves are more appropriate for fracture prediction. SEN(B) tests for fiber-reinforced concrete are prescribed in ASTM C1550 [118] “Standard Test Method for Flexural Toughness of Fiber Reinforced Concrete (Using Centrally Loaded Round Panel)”, ASTM C1609 [119] “Standard Test Method for Flexural Performance of Fiber-Reinforced Concrete (Using Beam with Third-Point Loading)”, and EN 14651 [120] “Test method for metallic fibered concrete—Measuring the flexural tensile strength (limit of proportionality (LOP), residual)”. For fracture characterization of regular non-reinforced concrete, the RILEM TC-50 FMC recommendation [121] is often used. RILEM does not issue standards like ISO or ASTM, but the RILEM Technical Committee (TC) Recommendations are used as a de facto standard for concrete fracture mechanics analysis and phase-field model calibration.

Although Single Edge Notch Tension (SENT) specimens share the notched-geometry family with SEN(B), they differ fundamentally in loading mode and application scope; they are introduced here for completeness but are not treated as a separate test category in this review. SENT specimens are primarily used to measure the fracture toughness of pipeline steels, as they best resemble the stress state in pipelines. SENT testing is performed according to the BS 8571 standard [122] “Method of test for determination of fracture toughness in metallic materials using single edge notched tension (SENT) specimens”. They are often used together with C(T) and SEN(B) to obtain more realistic and reliable predictions for industrial applications in pipelines.

**Figure 3:**
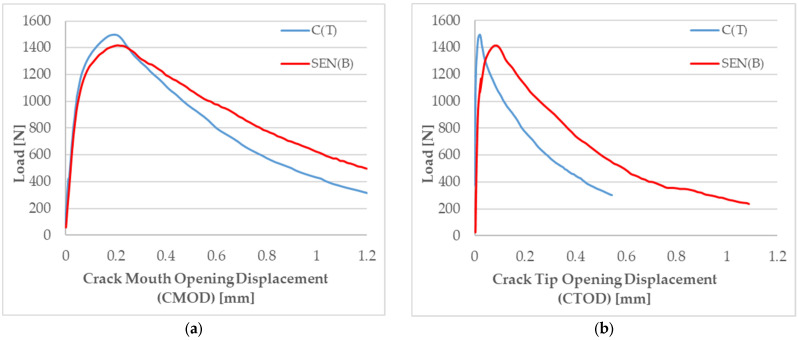
Experimental characterization of X52 pipe steel performed by Agbo et al. [123]: (**a**) load–CMOD; (**b**) load–CTOD.

Agbo et al. studied the fracture toughness of X52 pipe steel using C(T), SENT, and SEN(B) specimens, and concluded that the fracture toughness predictions from different tests are not directly correlated due to the different constraint levels [123]. SENT specimens are geometric variations of flat dog-bone specimens (with the added machined notch), but they serve entirely different purposes, i.e., obtaining fracture mechanics material characteristics like CMOD and CTOD, J-integral, and resistance to ductile crack extension (R-curve). As their application is narrowly focused on metal pipes, they will not be discussed in a separate chapter; instead, the focus will be on SEN(B) tests, which have more versatile coverage of materials studied by fracture mechanics. To showcase the difference in fracture response between C(T) and SEN(B) specimens, load–CMOD and load–CTOD diagrams were reconstructed from data digitized from Agbo et al. [123] and are presented in Figure 3.

The dimensions of the outlined tested C(T) and SENB specimens are proportional, with a crack ratio of 0.5. The load–CMOD comparison shows a similar response; the curves are well matched throughout loading and less matched in the post-peak regime. The load–CTOD comparison, however, tells a different story. The higher-constraint C(T) specimen reaches its peak load much faster than its SENB counterpart, and it has a steeper decline in the post-peak regime. SENB’s lower constraint allows significantly more crack-tip opening, while the higher-constraint C(T) specimen produces higher triaxiality at the crack tip. Higher triaxiality reduces local plasticity, which limits the deformation the crack tip can suffer before the specimen reaches its load-carrying capacity. Therefore, C(T) gives more conservative results for fracture toughness, giving engineers a larger safety margin. On the other hand, SENB specimens give a more realistic prediction, especially if the actual construction is less constrained. If regulatory compliance is required and if the worst-case scenario in the critical installations must be avoided, C(T) specimens must be used; otherwise, SENB specimens better reflect realistic service conditions. Agbo et al. [123] concluded that fracture toughness predictions from various tests are not directly correlated and that a unified approach relating constraint levels to fracture toughness is needed.

The recommended ratio between specimen thickness B and width of the specimen W according to the ASTM E1820 [88] and ISO 12135 [92] standards is 2, but these standards also allow the alternative ratios in the range of 1 ≤ W/B ≤ 4. However, using test specimens made of VL E36 hull steel with a nominal thickness of 50 mm, Kowalski showed that experiments using smaller (W = B) specimens yield lower CTOD values [124]. He stated that CTOD is not a material constant but depends on geometry and crack length, and that regulatory bodies should set different criterion values for different specimens used.

There are several experimental ways of determining CTOD, and Khor et al. compared the silicone replication method, DIC, and clip gauge measurements using SEN(B) specimens made from SS316 austenitic stainless steel [125]. They used a two-part silicone compound (Microset RF-101) for crack casting, a non-contact optical 3D deformation measuring system, GOM-ARAMIS, for DIC measurements, and standard clip gauges. They concluded that DIC measurements have good agreement with silicone replicas and that they are more conservative at large displacement. Also, CTOD values calculated from clip gauge CMOD data underestimated the actual crack tip opening at the large displacement. Moreover, the calculation of CTOD from clip gauge data can be performed quickly from standard test data, while the direct measurement, with silicone replicas or DIC, is time-consuming, destructive, and not always feasible in industrial settings.

In a subsequent paper, Khor et al. [125] compared various standard methods for CTOD calculation using specimens made of steels with different strain hardening [126]. The ASTM E1820 [88] and ISO 12135 [92] standards use different equations for the calculation of CTOD. The comparison by Khor et al. [125] showed that ISO 12135 [92] uses the same principle as the outdated British BS 7448 standard [127], which does not account for strain hardening of the material, and is accurate for medium and high strength steels but less accurate for steels with a lower yield-to-tensile ratio. ASTM E1820 [88], on the other hand, significantly underestimates CTOD for many higher-strength steels, so Khor et al. [125] concluded that the most accurate prediction comes from the Japanese WES 1108 standard [128] “Standard test method for crack-tip opening displacement (CTOD) fracture toughness measurement”. This versatile standard lacks global adoption. Although both ASTM E1820 [88] and ISO 12135 [92] underestimate CTOD, they remain suitable for industrial applications precisely because conservative estimates provide a safety margin. In fracture mechanics, it is safer to underestimate toughness than to overestimate it because if the material is assumed to be less tough than it really is, it leads to safer design.

Kawabata et al. proposed a new CTOD calculation formula that considers the variation of crack tip blunting due to strain hardening [129]. They introduced a new factor *f*, which accounts for the yield-to-tensile ratio (YR) of the material and the specimen thickness in order to correct the plastic component of CTOD. The FEM calibration of the correction factor was done simulating SEN(B) specimens with nine different thicknesses of B = 5, 10, 25, 30, 50, 75, 100, 150, and 200 mm, with YR values ranging between 0.6 and 0.98, for a constant tensile strength of 520 MPa.

Štefane et al. studied the effects of fixture configurations and different weld materials on J-integral values [130]. The studied BM is S690 QL high-strength low-alloy (HSLA) steel, and for WM, two materials were used: Mn4Ni2CrMo (MIG 90) for overmatched welds and G4Si1 (VAC 65) for undermatched welds. Štefane et al. [130] concluded that varying the configuration of fixtures (fixed oversized rollers) affects plastic factor *η_pl_*, geometry factor λ, and corresponding plastic factor γ_pl_, which can lead to overestimated fracture toughness. Štefane et al. [130] highlighted the importance of abiding by the standard ASTM E1820 [88] for fixture setup, or if that is not possible, proposed calculations of these factors with equations that account for the fixed rollers.

Tomerlin et al. performed an experimental and FEM analysis of fracture mechanics properties of different zones in the welded joint of S690QL1 HSS grade steel [131]. They used miniaturized tensile specimens to determine stress–strain curves for each characteristic weld zone (BM, WM, HAZ), and standard SEN(B) testing according to ASTM E1820 [88]. They concluded that the combination of experimental tensile and fracture tests provides the necessary material parameters for accurate numerical simulations of mechanical heterogeneity and crack behavior of the welded joint, which was validated by comparison of FEM results with DIC measurements of experimental crack growth.

Conventional CTOD tests prescribe that specimens like SEN(B) and C(T) are highly constrained, and they fracture due to the bending load, while real structures like pipelines or LNG tanks often experience tensile and membrane loading and are far less constrained. The research by Agbo et al. featuring SENT specimens [122] used for the analysis of pipeline steels [123] was already discussed, and now the fracture toughness of LNG tanks will be briefly covered. Since there is no dedicated standard specimen geometry for LNG tanks, Kim, Oh, and Kim used SEN(B) and custom Wide Plate (WP) specimens to mimic crack growth in real working conditions [132]. Their work presents a fitness-for-service (FFS) analysis of an LNG tank made of 9% Ni steel that operates at −196 °C. This research was conducted according to the FITNET procedure [133,134], and it follows ISO 27306 [135] “Metallic materials—Method of constraint loss correction of CTOD fracture toughness for fracture assessment of steel components”. Kim, Oh, and Kim first performed CTOD tests on SEN(B) and Wide Plate (WP) specimens. Then, they used FEA simulations to obtain the near-crack-tip stress/strain fields needed for the calculation of Weibull stress distributions. Using the CTOD values and Weibull stress, they determined the equivalent CTOD ratio β. Finally, they used corrected CTOD toughness in a failure assessment diagram (FAD) to show how constraint correction reduces conservatism.

FITNET was a European project that had a great influence on the field of fracture mechanics, but its procedure was never adopted as an ISO or EN standard. It is still often cited in scientific papers, and many aspects of the FITNET documents were later incorporated into newer releases of the British BS 7910 standard “Guide to methods for assessing the acceptability of flaws in metallic structures” [136]. The most comprehensive framework for evaluation of the structural integrity of in-service equipment was the result of collaboration between the American Petroleum Institute (API) and the American Society of Mechanical Engineers (ASME), who jointly created a rulebook: API 579-1/ASME FFS-1 “Fitness-For-Service” [137].

Moore and Pargeter studied the challenges in determining load line displacement (LLD) and CMOD needed for the calculation of J-integral using SEN(B) specimens made of SA302 Grade C Mn-Mo-Ni alloy steel, SA543 Grade B high-strength Ni-Cr-Mo alloy steel, and S355 C-Mn structural steel [138]. They argue that LLD gauges placed on the specimen can be affected by load-point indentation, friction effects, and specimen geometry changes during the test, while clip gauges used to determine CMOD are easier to attach, more accurate, and allow for more repeatable tests; therefore, they recommend that CMOD should be used for J-integral calculation.

The following subsection covers papers on plain concrete and fiber-reinforced concrete. These papers often lack references to international standards [118,119,120] and RILEM recommendations [121], so remarks will be made that specify whether the research is conducted according to a standard or not.

Wang et al. analyzed progressive failure of concrete by phase-field modeling and experiments [139]. Their experiments are performed on specimens with dimensions of 400 mm × 100 mm × 100 mm, while the notch is 20 mm deep. The analyzed material is plain normal-strength concrete made using 425-grade ordinary Portland cement, sand, and gravel with a maximum aggregate particle size of 25 mm. Wang et al. [139] did not explicitly specify adherence to standards, but their specimen geometry and testing methodology do not deviate from standards, and experiments are not the main focus of this paper. Instead, its main contribution lies in using a Python (v 2.7) script to generate a random aggregate distribution in an ABAQUS UEL subroutine for meso-scale modeling of concrete that features different material properties of matrix, aggregates, and interfacial transition zone (ITZ). Around coarse aggregate particles in concrete, a thin region, which is typically 20–50 μm thick, is formed as a boundary layer between aggregate and cement paste with higher porosity and weaker bonding, making it ideal for crack initialization and propagation. Wang et al.’s comprehensive phase-field model showed excellent agreement with experimental results for Mode I and mixed-mode failure numerical analysis [139].

Tang and Chen studied the nonlinear fracture behavior of plain concrete in the fracture process zone (FPZ) using two groups of concrete specimens: normal-strength concrete and high-strength concrete with respective compressive strengths of 40 MPa and 90 MPa [140]. SEN(B) specimens are evaluated according to RILEM recommendations [121] with dimensions of 710 mm × 150 mm × 80 mm and a span of 600 mm. They used the electronic speckle pattern interferometry (ESPI) technique to measure crack evolution and deformation of the beams. Using experimental results and FEM simulations featuring a cohesive crack model, they evaluated different tension softening curves for analyzed normal-strength and high-strength concrete.

Chen et al. performed three-point bending tests at four different loading rates of 0.0005, 0.005, 0.05, and 0.5 mm/s to study how the loading rate affects concrete fracture [141]. They used 700 mm × 150 mm × 100 mm specimens with a 60 mm notch made using 425-grade ordinary Portland cement. The experiments were performed according to RILEM recommendations [121], and load–CMOD curves were obtained from the clip gauge and DIC measurement. Numerical simulation was done using the Extended Finite Element Method (XFEM) model in ABAQUS. Chen et al. [141] concluded that the peak load and peak CMOD increase with an increase in loading rate. Also, the fracture characteristic length increases with an increase in loading rate, and so does the fracture energy and the brittleness. Crack path also changes at higher strain rate, as the cracks often do not have enough time to divert through the ITZ region, but instead, they propagate directly through coarse aggregates.

At the same time, Bu et al. also explored the influence of various loading rates on the propagation of FPZ [142]. They chose 0.001, 0.01, and 0.1 mm/s as loading rates, which they applied to 400 mm × 100 mm × 100 mm specimens with a 30 mm machined notch (referred to by the authors as a pre-crack). The specimens were made using 425-grade ordinary Portland cement, river sand, and aggregates with a maximum size of 20 mm. The proportions of concrete constituents are chosen according to ASTM C33/C33M [143] “Standard Specification for Concrete Aggregates”, while testing itself is done according to RILEM recommendations [121]. They used DIC to monitor COD and the evolution of FPZ, and concluded that the length of FPZ first increases and then decreases as the effective crack length grows, and that the maximum length of FPZ is about 60 mm. They also concluded that at higher loading rates, the FPZ length at peak load is shorter.

Xu et al. analyzed the boundary effect on the fracture energy determined according to RILEM recommendations [121] using 60 SEN(B) specimens of varying sizes, crack length-to-depth ratios, and span-to-depth ratios made from high-strength concrete that consisted of Portland cement, fly ash, silica fume, sand, and crushed stone [144]. They developed a bilinear local fracture energy distribution model, expressed as a piecewise function linking the experimental test fracture energy G_f_, the local fracture energy g_f_, and the fracture energy unaffected by specimen size G_F._ This model enables the calculation of fracture energy, unaffected by specimen size, from small-scale tests.

Similarly, Lehký et al. performed a comprehensive analysis of the dependence of mechanical fracture parameters on the size of the test specimens. Besides SEN(B) specimens tested according to RILEM recommendations [121], they also performed wedge-splitting tests (WSTs) on C30/37 grade concrete [145]. C30/37 is a concrete grade defined in EN1992-1-1 [146] “Eurocode 2: Design of concrete structures” that has a Cylinder strength of 30 MPa and a Cube strength of 37 MPa. WSTs are often found in scientific literature, and although they are not standardized by ASTM or ISO/EN standards, they are well defined in RILEM recommendations [121]. WST specimens somewhat resemble C(T) specimens and have a notch that is loaded via a wedge, converting vertical force into horizontal splitting force and providing stable crack propagation and reliable measurement of fracture energy and toughness, even for large specimens. Lehký et al. [145] used geometrically scaled specimens with varied notch depths, with special attention paid to the design of the most appropriate concrete mixture, namely, the selection of a maximum aggregate grain size. Using the effective crack-length formulation and work-of-fracture method, they evaluated fracture parameters at different stages of concrete aging and hardening.

Maulana et al. focused their research on foamed concrete, a lightweight, cost-effective mix with great thermal and acoustic insulation [147]. Foamed concrete has at least 20% air in its volume and no coarse aggregates, making it less dense than regular concrete, which comes at the cost of reduced compressive strength and load-bearing capacity. Foamed concrete is not suitable for structural elements but for void filling and insulation. Maulana et al. [147] made their specimens using 425-grade ordinary Portland cement, river sand with a maximum grain size of 3 mm, silica fume, and a synthetic-based foaming agent used to generate foam. Experiments included a four-point bending test on un-notched concrete beams according to ASTM-C78/C78M [148] “Standard Test Method for Flexural Strength of Concrete (Using Simple Beam with Third-Point Loading)”, a three-point bending test on SEN(B) specimens, and uniaxial compression tests on cylindrical specimens (that will be described in the next chapter). Maulana et al. [147] used DIC and strain gauges in their experiments, and XFEM with Cohesive Zone Model (CZM) for numerical simulations in ABAQUS. CZM features a gradual loss of material cohesion across an FPZ and is suitable for modeling of fracture initiation and growth in quasi-brittle materials, but it requires a predefined crack path, unlike the phase-field model, which allows for natural crack initiation, branching, and merging. Maulana et al. [147] used inverse analysis to determine the critical crack length and other material parameters by fitting a numerical model to match experimental load–CMOD curves.

Wang et al. proposed a localizing gradient damage model to predict the fracture of fiber-reinforced ultra-high-performance concrete (FRUHPC) [149]. The fibers used in the studied concrete are 13 mm long, made from polyethylene, and constitute 0.8% of the total volume. The specimens they used for the three-point bending test have dimensions of 500 mm × 100 mm × 100 mm, while the notch is 30 mm deep and 5 mm wide. Although Wang et al. [149] do not explicitly state that they followed any standard, their specimen geometry and experiment methodology do not deviate from standardized practices used in the fracture mechanics analysis of concrete beams. They used acoustic emission (AE) to monitor events in the FPZ, such as the emergence of numerous fine cracks and a slow widening of these active cracks due to the fiber bridging effect. After the initial linear elastic phase, strain hardening occurs, and afterwards, when a certain threshold limit is reached, the structure starts to strain soften gradually, as a result of fiber pullout and the formation of a localized macroscopic crack.

Similarly, Liang et al. studied the hybrid effect of coarse polypropylene fibers and basalt fibers on the fracture toughness of reinforced concrete [150]. They used AE for the determination of crack initiation and the double K fracture criterion to calculate toughness parameters. The concrete matrix grade is C50, and a total of 13 different fiber content combinations were tested. The mixing, pouring, curing, and testing were done according to national (Chinese) standards, with bars of 515 mm × 100 mm × 100 mm and a notch depth of 40 mm. Liang et al. [150] concluded that the optimum content of coarse polypropylene is 6 kg/m^3^, and for the basalt fiber, it is 3 kg/m^3^ (ratio 2:1). They also concluded that the bilinear softening constitutive curve proposed by Xu and Reinhardt accurately describes the fracture process of the hybrid-fiber concrete.

Sucharda studied steel-reinforced concrete using a comprehensive series of experimental procedures, including tri-point bending of SEN(B) specimens made from plain concrete and tri-point bending of un-notched reinforced specimens [151]. His specimens were made from “Baumit ProofBeton”, a waterproof, frost-resistant concrete mix (class C30/37) which is certified for contact with drinking water and resistant to de-icing salts. His focus was on the shear resistance of structural beams without shear reinforcement, so his un-notched specimens included 24 reinforced concrete beams 100 mm × 190 mm in cross-section and with a span of 900 mm. At the bottom side, two longitudinal reinforcement steel bars are incorporated into the concrete beam. The bars are made of B500 steel grade and have diameters of 6, 8, 10 and 12 mm. Sucharda proposed an inverse method that combines multi-criteria decision analysis (MCDA), stochastic modeling with Latin hypercube sampling (LHS), and nonlinear FEM analysis for the determination of fracture mechanical parameters. His model provides more reliable predictions of shear failure than empirical code formulas from Eurocode 2 [146].

### 2.4. Uniaxial Compression Tests

The simplest experimental tests of concrete, which are also performed on rock samples, are uniaxial compression (UC) tests. These tests are designed to measure the load-bearing capacity of concrete and rock under monotonic compression load, using cylindrical and prismatic specimens. In these tests, an increasing axial load is applied steadily to cylindrical or prismatic specimens until failure, while recording stress–strain curves, peak compressive strength, and post-peak softening behavior. The numerical analysis of dams requires material characterization for both the dam body and the surrounding rock mass; however, in this review, the focus is on the construction materials, and the rocks will not be discussed. The concrete has a nonlinear and quasi-brittle nature, which involves strain localization, microcracking, and progressive damage accumulation. Material parameters such as compressive strength, elastic modulus, and strain at peak stress are determined and used to calibrate constitutive damage models. UC tests are conducted according to ASTM C39 [152] “Standard Test Method for Compressive Strength of Cylindrical Concrete Specimens”, ASTM C469/C469M [153] “Standard Test Method for Static Modulus of Elasticity and Poisson’s Ratio of Concrete in Compression”, EN 12390 [154] “Testing of hardened concrete Part 3: Compressive strength of test specimens”, and ISO 1920-4 [155] “Testing of concrete Part 4: Strength of hardened concrete”. UC tests are key for practical strength assessment, as the compressive stress is the dominant loading mode in concrete structures, and in that role, prisms/cubes are common construction practice for routine strength checks, but they have higher lateral restraint at platen–specimen contact. Cylindrical specimens are more accurate, i.e., have less scatter in modulus and post-peak data, so they are preferred for research and modeling but are more difficult to prepare.

The most detailed recent analysis of overcoming the disadvantages of prismatic and cubic specimens was performed by Bandeira et al., who assessed the influence of the boundary conditions and specimen geometry on material parameters in unconfined compression tests [156]. They used cylindrical specimens with a diameter of 100 mm and a height of 200 mm, prisms with dimensions of 100 mm × 100 mm × 200 mm, and 100 mm × 100 mm × 100 mm cubes, made from two grades of concrete, C50 and C30. Their study included five boundary conditions: grease, Teflon, brush plate, glued sheets, and regular contact. The strength difference between prisms and cubes was, on average, 15% for C50 and 35% for C30 concrete, while the differences using cylindrical and prismatic specimens with the same slenderness were, in all cases, negligible. No significant difference was found between cylinders and prisms, confirming that geometry effects vanish when friction is reduced. Their experiments agree with the hypothesis that post-peak softening is not a characteristic of the material but rather a consequence of the interface friction in the tests. Additionally, the cubic specimens, which were provided with grease to eliminate friction, yielded the same compressive strength as cylinders or prisms with the same cross-sectional area. Bandeira et al. [156] highlighted the cylindrical specimens’ advantage in easy determination of lateral strains using a linear variable differential transformer (LVDT), while the prismatic specimens allow the use of DIC. Cubic specimens, on the other hand, allow the experimental determination of the strengths in the direction of casting or in orthogonal directions, enabling the study of anisotropy, which, according to Bandeira et al. [156], may reach around 10%.

Ivanchev studied the accuracy of non-destructive testing methods: elastic rebound (Schmidt hammer), Ultrasonic Pulse Velocity Method (UPVM), and SonReb, which is an analytical method based on the results of rebound and UPVM measurements [157]. For comparison, as a gold standard, Ivanchev performed uniaxial testing according to EN 12390 [154] on 150 mm cubes, and cylinders with a height and diameter equal to 100 mm. What sets his research apart is the fact that he performed his experiments after 28, 244, 280, 293, 342, 1126, and 1926 days, following the changes in the characteristics of concrete occurring with time. Changes in the compressive strength of concrete happen due to various factors, such as aging, temperature changes, shrinkage, environmental action, changes in humidity, poor or improper service and maintenance. Ivanchev concluded that the SonReb method yields the most accurate prediction, with a relative error between 0 and 4.6% in comparison to the destructive measurement, followed by the UPVM method with a relative error ranging between 0.3% and 9.6%. The relative error when using the Schmidt hammer ranged from 0 to 14.1%.

Lee et al. investigated the effects of cylinder size on the determination of static and dynamic elastic modulus and compressive strength for different grades of concrete [158]. They used 240 cylindrical specimens, 120 with a diameter of 100 mm and a height of 200 mm, and 120 with a diameter of 150 mm and a height of 300 mm, made from three concrete grades with compressive strengths of 30, 35, and 40 MPa. They performed static and dynamic tests 4, 7, 14, and 28 days after molding. Based on the experimental results, conducted according to the ASTM C469/C469M standard [153], they concluded that for normal-strength concrete (≤40 MPa), the two different cylinder sizes yield similar results for compressive strength and static and dynamic elastic modulus. However, they observed that the size effect became significant in high-strength concrete with a compressive strength over 40 MPa. Therefore, they recommended special care when comparing the static and dynamic properties of high-strength concrete using data from the two different cylinder sizes.

Miazgowicz and Domagała’s initial focus was on the scale effect on the mechanical properties of high-strength concrete [159]. Motivation for their work lies in the fact that the European standard EN 12390 [154] allows different procedures and different shapes and sizes of test specimens, but it does not provide a relationship between specimen size and shape and elastic modulus. They analyzed prismatic (inaccurately designated as cubic) and cylindrical specimens of various sizes and slenderness, machined from prefabricated high-strength concrete elements supplied by a private manufacturer who did not provide details about the concrete mix. The reference geometry prescribed by EN 12390 [154] is a cylinder of 150 mm in diameter and 300 mm in height; Miazgowicz and Domagała also studied cylindrical specimens with diameters of 80 and 100 mm, and prismatic specimens with widths of 80, 100, and 150 mm, with heights ranging from 160 to 400 mm for both cylindrical and prismatic specimens. Their experiments showed no significant scale effect in either the elastic modulus or compressive strength tests. Miazgowicz and Domagała concluded that the differences in the density of individual specimens make it difficult to identify the scale effect in the case of the compressive strength test, and that individual specimen structure (porosity) plays a much bigger role in comparison to the scale effect.

It is worth noting that the results of Miazgowicz and Domagała [159] contradict those of Lee et al. [158], who found that for normal-strength concrete (≤40 MPa), two standard cylinder sizes (150 mm × 300 mm and 100 mm × 200 mm) yield similar results, while the size effect becomes significant in high-strength concrete exceeding 40 MPa. Miazgowicz and Domagała [159] found no significant scale effect in concrete with a compressive strength of 101.9 MPa, well above Lee et al.’s [158] 40 MPa threshold, leaving this contradiction an open question in the literature.

Wang et al. investigated the role of boundary friction between the concrete cube specimens (50 mm × 50 mm × 50 mm) and loading platens [160]. The focus of their research was the variability of mechanical response, scatter of the results, and localized differences that are the consequence of the random distribution of aggregates, cement, ITZ, and voids. Experiments were performed on concrete specimens, made from ordinary Portland cement (P.I. 42.5) with coarse aggregates (3–12 mm) and river sand and cured for 28 days. They developed a 3D meso-scale FEM model and simulated uniaxial compression loading with a contact friction coefficient ranging from 0.0 to 0.7. Their analysis showed that the frictional contact had a limited influence on the elastic compressive mechanical behavior of concrete, but the nonlinear hardening behavior of the stress–strain curves showed a significant relation with the frictional contact.

Tasevski et al. investigated the development of linear and nonlinear creep strains and concrete failing mechanisms under high stress levels [161]. The analyzed material is normal-strength concrete, composed of CEM-II 42.5R cement (water to cement ratio w/c = 0.56) and Rhone River aggregates. Tasevski et al. [161] used cylindrical specimens with a diameter of 160 mm and a height of 320 mm, which were kept in the mold for 21 days. First, they varied the strain rate, using specimens aged between 10 and 14 months to limit the strength increase effects. In the second series, the authors varied the stress rate, using specimens aged between 22 and 24 months. The longitudinal strain of the specimens was measured with displacement transducers (omega gauges), while the transverse strain was measured with a dilatometer with an LVDT. The prolonged high stress levels have a significant impact on the compressive strength and deformation capacity of concrete. Tasevski et al. [161] concluded that concrete strength under long-term loading correlates with the inelastic strain capacity of concrete, and they developed a failure criterion based on this property.

Czajkowska et al. determined the linear correlation coefficient between Young’s modulus and the compressive strength for two grades of fiber-reinforced concrete with steel fiber content of 0.25% and 0.50% [162]. Since concrete does not have a linear stress–strain relationship, the initial tangent modulus is greater than the tangent modulus at service load, so according to EN 12390 [154], a secant modulus is calculated after three preloading cycles, with the nominal upper stress σ_a_ taken as 1/3 of compressive strength *f*_c_, while the nominal lower stress σ_b_ has an arbitrary value between 10% and 15% of compressive strength *f*_c_. Calculation of Young’s modulus for concrete is difficult due to the concrete’s heterogeneous nature and uneven distribution of aggregates, cement paste, pores, and fibers, which can cause scatter in strain readings, so Czajkowska et al. [162] used linear regression to obtain the coefficient of linear correlation. They used 16 cylindrical specimens with a diameter of 150 mm and a height of 300 mm made using C30/37 grade concrete with w/c = 0.43. Steel fibers with a length of 60 mm and a diameter of 1.0 mm (commercial BauMix 60/1 type) were used to reinforce the concrete matrix. Their research showed a significant correlation between compressive strength and Young’s modulus, with the correlation coefficient r and coefficient of determination r^2^ for specimens containing 0.25% of steel fibers being r = 0.8823 and r^2^ = 0.7785, and for specimens containing 0.50% of steel fibers, coefficients r and r^2^ are even higher with r = 0.9862 and r^2^ = 0.9726.

Ferrotto et al. analyzed structural retrofitting procedures using Carbon Fiber-Reinforced Polymer (CFRP) wraps [163]. Since concrete columns are often strengthened under serviceability load conditions, the stress and strain state differ in comparison to the unloaded state, which is extensively studied. Therefore, Ferrotto et al. [163] developed a model that takes into account the presence of an existing stress/strain state in a concrete column at the moment of application of the fiber wraps. Analyzed concrete has unconfined compressive strength between 38.13 MPa and 41.7 MPa and an elastic modulus between 32,586 and 35,253 MPa. The specimens tested by Ferrotto et al. [163] had a diameter of 150 mm and a height of 600 mm, which is a non-standard 1:4 ratio. Higher slenderness of specimens means they are more prone to buckling and susceptible to lateral strain effects but have a more accurate representation of the behavior of real structural columns. Ferrotto et al. [163] used unidirectional carbon fibers with an elastic modulus of 234 GPa and a nominal thickness of 0.131 mm for the reinforcement sheets. They applied three different preloading levels: 40–55%, 60–70%, and 80–90% of the unconfined compressive strength of concrete. Based on the experimental results, Ferrotto et al. [163] proposed a constitutive model for preloaded FRP-confined concrete that reproduces both ultimate conditions (confined strength and strain) and the reduction in secant stiffness dependent on the preload level.

Xu et al. studied cyclic stress–strain behavior of blended fiber-reinforced concrete (BFRC) that contains a combination of steel and polypropylene fibers [164]. They used prismatic specimens with dimensions of 150 mm × 150 mm × 300 mm made from concrete reinforced with steel and polypropylene fibers to perform cyclic compression experiments according to national (Chinese) standards. The plain concrete matrix was made using Portland cement P.O. 42.5 as the binder, coarse aggregates composed of crushed granitic rocks with a maximum size of 20 mm, and fine river sand. Steel fibers (SFs) with an aspect ratio between 30 and 80 constitute volume fractions in concrete of 1.0%, 1.5%, and 2.0%. Polypropylene fiber (PF) with an aspect ratio of 167, 280, and 396 constitutes volume fractions in concrete of 0.1%, 0.15%, and 0.2%. Based on experimental results, Xu et al. [164] developed a semiempirical constitutive model for BFRC under uniaxial cyclic compression. This study shows that blended fibers improve peak strength and post-peak ductility and decrease plastic strain accumulation compared to plain concrete.

Schlappal et al. studied the creep and cracking of concrete hinges [165]. These specific construction elements are key components of many bridges and represent a durable, maintenance-free alternative to mechanical steel bearings. They are created by planned reduction of the cross-section of a concrete column designated as “hinge neck” or “throat” that concentrates compressive stresses in a narrow zone, creating a state of multiaxial loading, which allows large rotations. They are monolithic, i.e., cast as part of the structure, and contain little or no steel reinforcement. Schlappal et al. [165] performed centric and eccentric compression experiments, according to national (Austrian) testing standards and Leonhardt–Reimann hinge guidelines. They used plain concrete prisms and marginally reinforced hinge specimens made from C 35/45 F45 GK16 B5 grade concrete made using CEM II/A-L 42.5 cement and calcite aggregates with a maximum size of 16 mm and w/c ratio of 0.48. Tested prisms have dimensions of 75 mm × 75 mm × 250 mm, while hinge specimens are 250 mm × 350 mm × 400 mm with B550A steel reinforcements. Lateral notches are 87.5 mm deep, while the front notches are 50 mm deep. Crack propagation was monitored with DIC, and rotations were measured with LVDTs. Their experiments showed that structural creep under eccentric compression is significantly amplified by bending-induced crack propagation. Based on these findings, to avoid progressive damage under permanent compressive normal forces, Schlappal et al. [165] recommended that permanent compressive normal forces should be limited to 45% of the corresponding ultimate load-carrying capacity of the concrete hinge.

Dong et al. developed a piecewise damage constitutive model that describes the concrete–rock interface [166]. Their model is not a general-purpose rock or concrete model; instead, it is specifically designed to improve the accuracy of the interface zone simulation, taking into account energy evolution, which is not available in FEM contact definition. Their piecewise model separates the compaction stage, where microcracks and pores close, from the damage stage, where stored energy is released and dissipated. Dong et al. [166] performed uniaxial testing on 40 specimens with various combinations of concrete component height, interface inclination angle, and coarse aggregate contents. The specimens had a diameter of 50 mm and a height of 100 mm. The analyzed granite has a uniaxial compressive strength of 116.52 MPa and an elastic modulus of 22.73 GPa, while the concrete portion of the specimen has a uniaxial compressive strength of 46.66 MPa and an elastic modulus of 18.87 GPa and is made from ordinary Portland cement (P.O 42.5), coarse aggregates with a size between 5.0 and 12.0 mm, and river sand. Molding and curing were done in accordance with national (Chinese) regulations, while the experiments were recorded using an axial LVDT and 3D laser scanner. Based on experimental results, Dong et al. [166] identified distinct failure modes and energy conversion mechanisms, which they incorporated into their material model.

The greatest deficiency of concrete is its low tensile strength, which is so pronounced that it is typically ignored in the design of concrete elements. However, this does not mean the researchers ignore this property. Badarloo et al. used the Brazilian test, also known as the splitting tensile test, to measure the tensile strength of concrete [167]. In this test, the cylindrical concrete specimen is placed laterally on the testing machine, and diametrical compressive force is applied along the side of the cylinder until it splits in half. Badarloo et al. [167] used this technique on 42 cylindrical specimens of 300 mm × 150 mm with 42 cubic specimens of 150 mm × 150 mm × 150 mm used for measurement of compressive strength. The specimens were made from C20, C30, and C40 concrete grades at curing ages of 7 and 28 days. They showed that the tensile strength increases with the compressive strength of concrete, more at 28 days than at 7 days.

Furthermore, Nilimaa and Nilforoush proposed, tested, and evaluated a general method for the direct tensile strength of concrete [168]. Their paper could have been placed in the uniaxial tensile testing section of this review, but due to the specimen geometry and testing procedure, it belongs in uniaxial compression tests, even though the loading is in reverse. Nilimaa and Nilforoush used a total of 16 cylindrical specimens cast in steel molds using a normal-weight concrete with a w/c ratio of 0.55. The specimens have a diameter of 100 mm and lengths of 100 and 200 mm. Nilimaa and Nilforoush then cut circumambient grooves, 10 or 15 mm deep, at mid-height of the cylinder specimen for crack initiation and monitoring. They used a two-component epoxy adhesive, HBM X 60 Epoxy, to glue the cylinder samples to steel plates. These steel plates are fastened to the testing machine grips with six bolts. Six additional cubes (100 mm × 100 mm × 100 mm) were used to measure the compressive and tensile-splitting strength. The experiments yielded an average tensile strength of 3.81 MPa and an average tensile-splitting strength of 5.55 MPa for the tested concrete. Nilimaa and Nilforoush concluded that the method is reliable, but more tests are needed for statistical validation. They emphasized its potential for structural health monitoring and improving empirical relations for the tensile strength of existing concrete.

The production of green, sustainable concrete with recycled waste materials has gained a lot of attention in recent years. Alani et al. studied ultra-high-performance concrete made with ultrafine palm oil fuel ash (UPOFA), which replaces 20–40% of cement, and 1% PET fibers, tested on cube (100 mm), cylinder (100 mm × 200 mm), and prism (100 mm × 100 mm × 500 mm) specimens [169]. They performed compressive strength tests according to EN 12390 [154], but also porosity, permeability, and chloride (NaCl) penetration tests. They concluded that combining agro-waste UPOFA and recycled PET fibers yields sustainable UHPC with enhanced strength and transport properties. Based on their experimental results, the concrete that has 20% of Ordinary Portland cement replaced with UPOFA has the greatest increase in compressive strength, while the concrete with 40% UPOFA replacement has the lowest porosity, permeability, and chloride ion penetration.

Chen et al. tested fly ash concrete under sustained compressive load [170]. Since external loads can accelerate the hydration of cement, this can lead to an increase in the strength and the elasticity of the loaded concrete. Although this concept is over a century old, the incorporation of supplementary cementitious materials (SCMs) and waste/by-products for sustainability introduced additional complexity to the time-dependent behavior of concrete. Chen et al. [170] used two different sizes of a Representative Volume Element (RVE) to represent the micro-heterogeneous space of binder and concrete. Their specimens were made using Ordinary Portland Cement P.O 42.5, with fly ash replacement ratios of 20% and 40%. Specimens were prism blocks 100 mm × 100 mm × 300 mm, tested according to local (Chinese) standards. The elastic modulus was measured after 7, 28, 60, 90, 120, and 180 days of curing. Chen et al. [170] applied the sustained load using a self-fabricated spring-reaction device and concluded that concrete with a lower fly ash replacement ratio of 20% has a greater elastic modulus at all ages, and that sustained load increases the elastic modulus for both replacement ratios up to 5% of the non-loaded concrete.

Another example of eco-friendly concrete is gangue concrete, studied by Xiao et al. [171]. Unlike gravel and river sand, whose exploitation has caused serious damage to the river’s ecological environment, gangue is an abundant solid waste produced in coal mining that can be crushed into appropriately sized aggregates for concrete [171]. Xiao et al. [171] used cube specimens 150 mm × 150 mm × 150 mm made from both coal gangue aggregate concrete and regular sand/gravel concrete to perform uniaxial compression tests with AE monitoring according to national (Chinese) standards. Based on experimental results, they found that gangue concrete achieves 35–40 MPa strength but has higher porosity, brittleness, and abrupt rupture compared to ordinary concrete. AE analysis revealed two cumulative energy peaks before failure, unlike the single peak in conventional concrete. Xiao et al. [171] concluded that gangue is a sustainable alternative aggregate, and AE technology can also be used for predicting damage in gangue concrete structures.

Zhang et al. investigated the influence of fiber content, sustained stress levels, and specimen age on the creep behavior of polypropylene fiber-reinforced alkali-activated slag (FRAAS) concrete [172]. This concrete is not made using Portland cement; instead, the binder is ground granulated blast furnace slag (GGBFS), an industrial by-product from steelmaking. They performed testing on cylindrical concrete specimens (100 mm × 200 mm) with fiber dosages of 0%, 0.5%, and 1.0%, according to national (Chinese) standards. The authors identified a damage threshold in alkali-activated matrices where microcrack propagation shifts from matrix densification at low stress to destructive coalescence at higher stress. Fiber reinforcement constrained microcrack growth and delayed macrocrack formation by redistributing localized stresses.

Kirthika and Singh researched properties of concrete specimens made from recycled fine aggregates (RFAs) obtained from construction and demolition waste and performed experiments at 7, 28, 56, and 90 days of curing for each mix (Control, RFA30%, RFA50%, RFA75%, RFA100%) [173]. They performed uniaxial compression tests, as well as splitting and flexural strength, according to the local (Indian) standard at a loading rate of 0.5 mm/min using 150 mm cubes, 150 mm × 300 mm cylinders, and 100 mm × 100 mm × 500 mm prisms. Kirthika and Singh’s work focuses mostly on shrinkage, permeability, chloride penetration, carbonation, resistivity, and chemical exposure, which they studied according to international standards, but this review is dedicated to mechanical characterization, so in that regard, they concluded that the RFA30% mixture has the greatest compressive strength for all curing ages.

Another branch of industry that has gained a lot of popularity in recent years is the 3D printing of cementitious materials. Skibicki et al. studied the experimental determination of Young’s modulus for hardened 3D-printed mortar [174]. Unlike concrete, the mortar does not include coarse aggregate, so it is easier to pump and print, but it is generally weaker. Skibicki et al. [174] used a mortar mix composed of CEM I 52.5R cement, fly ash, silica fume, fine sand aggregates (0–2 mm), and a polycarboxylate admixture. They used four types of cylindrical specimens; the first type was mold-cast, the second was printed within the mold. The third type of specimens was created by printing columns, and the fourth type was prepared by cutting them from a bigger 3D-printed multi-layer block. The first three types had a diameter of 150 mm and a height of 300 mm, while the diameter of the cut-out samples was 44 mm, 74 mm, 99 mm, and 144 mm, based on available core drill sizes. Skibicki et al. [174] concluded that the strength reduction compared to the standard cylindrical sample was the highest for freely printed columns (approximately 43%) and the lowest for samples printed into a mold or cut out from a bigger printed block. Skibicki et al. [174] identified the lack of an appropriate standard method for determining material parameters of 3D-printed concrete for real-life structural applications.

**Figure 4:**
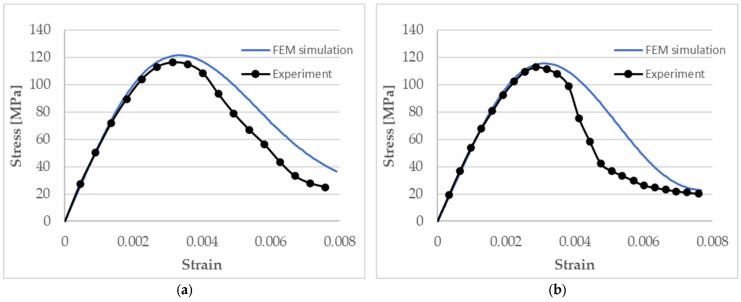
Stress–strain curves from experiment and FE modeling performed by Zhu et al. [175]: (**a**) CNTs 0.08%, steel fibers 2%; (**b**) CNTs 0.5%, steel fibers 2%.

The most high-tech solution of concrete reinforcement implies the incorporation of carbon nanotubes (CNTs). Zhu et al. [175] used 36 cylindrical specimens (50 mm × 100 mm) made from CNT-reinforced concrete with graphene oxide (GO) dispersant and optional steel fibers, fabricated and tested according to ASTM C39 [152]. The mass fractions of CNTs were 0, 0.08%, and 0.5%, while the steel fiber content was measured as a volume fraction, i.e., 0 or 2%. Based on the experimental results, they concluded that CNTs had a limited influence on failure mode and elastic modulus but modestly improved peak stress and strain at a lower fraction (0.08%). Steel fibers, however, significantly increased peak stress, peak strain, elastic modulus, and toughness index, transforming brittle failure into ductile behavior. However, Zhu et al. [175] found a significant discrepancy between the test and the FEM simulation in the descending section of the stress–strain curve, as can be seen in Figure 4.

They attributed these differences to defects present in the test specimens compared with the ideal condition in the simulation and also concluded that properties of CNTs at the nanoscale need to be further studied.

### 2.5. Literature Search Methodology

To complement conventional database searches, an AI-assisted literature discovery tool was employed. Undermind.ai [176] was selected for its superior retrieval performance in the field of experimental mechanics of construction materials.

The following is an example of a full search query used in Undermind: “I want to find recent (last 10 years), high-impact experimental mechanics papers in which clearly standardized uniaxial compression (UC) tests are a central experimental component, with detailed documentation of test setup and protocol (including standards used) for plain and reinforced concrete of any type, and I want these papers prioritized by citation count”. That query yielded 188 papers, from which the 40 most relevant papers were extracted. The relevance is based on “Topic Match” metrics by Undermind. The extracted papers were then carefully read, and the most appropriate for this review paper were chosen. A total of 20 papers on UC testing are reviewed, organized progressively from standard testing procedures and specimen geometry studies, through fiber-reinforced and structurally specialized configurations, to sustainable concrete alternatives and emerging high-tech reinforcement approaches.

## 3. Results and Discussion

This review paper covers the most used and standardized experimental procedures for testing construction materials, with a focus on steel and concrete, and features articles published in the last decade that demonstrate innovation and advance the state of the art. The body of experimental work surveyed in Section 2 reveals that the choice of test type is guided by its distinct role in the experimental characterization of construction materials. In general, only SEN(B) testing is equally applicable to both steel and concrete, while UT, C(T), and UC testing are material-specific. The only case in which two tests are interchangeable is fracture characterization of steel, which can be studied using either C(T) or SEN(B) experiments. Each of the four procedures is shaped by the structural application it serves, the failure mode it targets, and the constraints imposed by available material and equipment, and each exhibits a recognizable pattern of beyond-standard adaptation driven by specific research needs. Table 1 and Table 2 synthesize the evidence collected across Section 2: Table 1 maps each test to its specimen geometry, loading mode, key measured parameters, and documented advances beyond standard practice, while Table 2 translates that evidence into comparative guidance, i.e., when each test should be preferred, what its practical advantages and limitations are, and what drives researchers to deviate from standardized procedures.

The standardized testing procedures are used to ensure reproducibility and comparability of experimental results between researchers, engineers, and commercial vendors. Some standards are comprehensive, describing multiple specimen geometries and loading procedures, like ASTM E399 [86] for instance, while the others are very specific, like BS8571 [122], used to measure the fracture toughness of pipeline steels using unique SENT specimens. Most of the testing was done according to international standards or standards that have international adoption, like ASTM standards, but there is also a number of papers in which research was done according to national standards. The complete list of the international standards that guided experiments in the reviewed papers is shown in Table 3.

Although most of the experiments are performed according to standards, there are also many cases in which the authors used non-standard specimens or procedures. The most common are miniaturized specimens, which are an economically more feasible alternative to full-sized specimens, and are used, for example, in rapid alloy prototyping and can also facilitate hydrogen embrittlement studies. Another common reason for experimental testing using a non-standard specimen is material modeling, which requires, for example, center holes, notches, or grooves to be cut into specimens in order to change the stress state from uniaxial tension to multiaxial (triaxial) stress. Finally, the third common reason for non-standard dimensions is accurate representation of the behavior of real structures, like higher slenderness of cylindrical concrete specimens, which makes them more prone to buckling and susceptible to lateral strain effects, just like the actual bridge columns.

These deviations from standardized procedures inevitably raise questions of comparability. Miniaturized specimens, while practical, have documented limitations: Zhang et al. [44] showed that specimens with a width-to-thickness aspect ratio below 5 fail to capture post-necking behavior and that fracture angle and stress triaxiality are sensitive to size and geometry. Non-standard geometries designed to alter the stress state, such as notched, grooved, or shear specimens [56], produce results that are by design not directly comparable to standard uniaxial tests; their value lies precisely in expanding the range of stress states covered, at the cost of requiring additional interpretation and, typically, numerical support to extract meaningful material parameters. Finally, non-standard dimensions motivated by structural representation, such as the high-slenderness concrete cylinders used by Ferrotto et al. [163], introduce size and boundary effects that standard procedures are specifically designed to suppress. Researchers using such results should apply appropriate size-effect corrections or clearly document the deviation and its expected influence on measured parameters.

Some material characterization still requires standardization; for example, Nilimaa and Nilforoush proposed a direct tensile strength testing method for concrete from existing structures [168]. The key aspect of their work that requires formal regulation is specimen preparation and connection to the testing machine. Skibicki et al. pointed out the need for a single, standard method for determining material parameters of 3D-printed concrete for real-life structural applications [174]. Zhang et al. noted the absence of standardized design codes for alkali-activated concrete [172].

Depending on the nature of the experiment, the researchers used various measuring equipment ranging from load cells integrated into the testing machine; extensometers that are attached to the specimen gauge length to measure elongation; clip gauges (specialized extensometers used to measure displacement between two points, like CMOD, for instance); strain gauges, which are electrical resistive sensors bonded directly to the specimen surface; and LVDT transducers, which convert small linear displacements into electrical signals using a transformer principle; as well as acoustic methods for crack initiation and propagation monitoring and optical methods, namely DIC, for full-field contactless strain mapping.

Several cross-cutting observations emerge from the surveyed literature. DIC has become the dominant strain measurement technique across all four test types, progressively replacing contact extensometry, clip gauges, and strain gauges in recent publications, which is a trend driven by its full-field capability and compatibility with both standard and non-standard specimen geometries. A notable inconsistency in the literature concerns size effects: while size effect correction methods are well-established for SEN(B) concrete testing and miniaturized C(T) specimens for reactor steels, no equivalent consensus exists for miniaturized UT specimens or non-standard UC slenderness ratios, and comparability between studies using different specimen sizes remains an open question. Another recurring gap is the characterization of welded joints: cross-weld specimens and weld-region C(T) and SEN(B) tests appear frequently, yet the results are rarely cross-validated between test types, leaving the relationship between tensile and fracture properties across weld zones incompletely resolved.

The macroscopic UC stress–strain response of concrete is not only governed by the cement paste matrix but is also strongly influenced by the geometry, volume fraction, and interfacial properties of its constituent phases [139]. Under compressive loading, the shear and compaction displacements at aggregate–matrix interfaces govern the onset of meso-scale cracking, and their magnitude is directly controlled by the mixture design and ITZ quality. The mixture design includes the selection of aggregate gradation, fiber type, and fraction, as well as supplementary cementitious materials. This choice directly controls the ITZ properties and therefore the shape of the macroscopic stress–strain curve: peak strength, strain at peak, and the slope of the post-peak softening branch. For instance, the inclusion of steel or polypropylene fibers not only increases peak strength and ductility (as shown by Xu et al. [164] and Liang et al. [150]) but also modifies the post-peak energy dissipation mechanism by bridging meso-scale shear planes and delaying the coalescence of distributed microcracks into a localized failure band. Similarly, eco-friendly aggregate substitutes such as recycled fine aggregate (Kirthika and Singh [173]) or gangue concrete (Xiao et al. [171]) alter the compaction and shear displacement at aggregate interfaces under load, producing characteristic changes in UC curve shape that must be accounted for when using compressive test data to calibrate constitutive damage models. Researchers developing or calibrating UC-based damage models like meso-scale FEM approaches should report not only the measured macroscopic parameters but also the mixture design, aggregate gradation, and ITZ characterization data to enable meaningful cross-study comparison.

Finally, the reviewed literature reveals an asymmetry between steel and concrete research: steel testing has advanced substantially in the direction of multiaxial stress-state characterization, while concrete testing beyond standard advances is concentrated in structural representation and fiber reinforcement, with comparatively little attention to stress-state effects in concrete fracture specimens.

## 4. Conclusions

This review systematically surveyed experimental characterization of steel and concrete across four standardized test specimen types—UT, C(T), SEN(B), and UC—with a focus on how recent literature advances beyond what the governing standards prescribe. The survey reveals that SEN(B) is the only procedure equally applicable to both materials, and that the sole methodological overlap exists in steel testing between C(T) and SEN(B) for fracture characterization, which is resolved in practice by constraint considerations: C(T) is preferred for high-constraint fracture-critical applications, while SEN(B) better represents low-constraint structural scenarios such as beams and welded joints. Non-standard adaptations cluster around three recurring drivers: material shortage, stress-state enrichment, and structural representation, but their implications for result comparability and parameter extraction are not yet uniformly addressed in the literature, representing an open area for standardization efforts.

Reviewed papers were published in the last decade, and while many follow international standards, others deviate with a clear purpose, mainly falling into the categories of: miniaturized specimens due to material shortage, non-standard geometries for multiaxial stress-state calibration, and modified dimensions for accurate structural representation.

The survey of these papers reveals several unresolved issues that must be addressed to advance the field from fragmented empirical practice toward a coherent, standardized framework:

1. Size effect and comparability. Size-effect correction is well established for SEN(B) concrete testing and miniaturized C(T) specimens for reactor steels, yet no equivalent consensus exists for miniaturized UT specimens or non-standard UC slenderness ratios. Establishing universally accepted correction protocols or, where necessary, dedicated standards for miniaturized geometries is a prerequisite for reliable cross-study comparison and database-driven material modeling.
2. Material modeling with variation of stress state. A recurring pattern across both UT and C(T) testing is the deliberate use of non-standard specimen geometries to expand the range of local stress states beyond what standard specimens can provide. Multiaxial damage criteria such as Johnson–Cook and Bai–Wierzbicki require calibration data across a prescribed range of stress triaxiality and Lode angle combinations that no single standard specimen geometry can cover. In UT testing, notched, grooved, shear, and central-hole flat specimens, as well as notched and grooved cylindrical specimens, are used to generate controlled combinations of stress triaxiality and Lode angle required for the calibration of multiaxial damage criteria. In C(T) testing, specimens with machined side grooves, holes, and modified crack-plane orientations serve an analogous purpose: probing constraint variation at the crack tip to characterize fracture behavior under conditions that differ from the high-constraint standard geometry. Despite the prevalence of these approaches across the reviewed literature, the specific geometries used vary between laboratories and are not standardized. A coordinated, standardized test matrix for stress-state coverage in UT testing, analogous to what ASTM E8 or ISO 6892-1 provides for standard uniaxial conditions and equivalent guidance for non-standard C(T) variants, would represent a significant step toward comparability of multiaxial calibration datasets across institutions.
3. Weld zone characterization. Cross-weld UT, as well as weld-region C(T) and SEN(B) tests, appear frequently in the literature, yet results across test types are rarely cross-validated, leaving the relationship between tensile and fracture properties across BM, WM, and HAZ zones incompletely resolved. A coordinated round-robin study across test types for a representative structural steel welded joint would fill this gap.
4. Concrete beyond compression. UC testing dominates concrete characterization, yet the direct tensile strength of concrete remains poorly standardized for in situ core extraction. Similarly, 3D-printed concrete and alkali-activated concrete currently lack dedicated standard test methods, creating barriers to structural qualification.
5. Measurement technology integration. DIC has emerged as the dominant non-contact measurement technique, yet its use is not yet formally incorporated into most governing standards for the four test types covered here. Standardizing DIC-based virtual extensometry, including speckle pattern requirements, camera calibration, and strain calculation algorithms, would enhance reproducibility across laboratories.

Addressing these issues requires coordinated effort between standards bodies (ASTM, ISO, RILEM), research institutions, and industry stakeholders.

In future work, this experimental characterization framework will be expanded to aluminium and composites, as well as to geomechanical testing of rocks and soils, guided by the practical demands of engineering analysis and FEM software development.

## Figures and Tables

**Table 1:** Synthesis of experimental procedures: Specimens, standards, measured parameters, and modeling relevance.

Test Type	Uniaxial Tensile—UT	Compact Tension—C(T)	Single Edge Notched Bending—SEN(B)	Uniaxial Compression—UC
Material	Steel (primary)	Steel	Steel & Concrete	Concrete (primary)
Specimen geometry	Dog-bone, flat or round bar specimens with uniform gauge section	Compact specimen with a machined notch and loading holes	Rectangular beam with single-edge notch, loaded in three-point bending	Cylindrical or cubic specimens under axial compression
Loading mode	Uniaxial tension to failure	Cyclic/monotonic tension on pre-cracked specimen	Three-point bending on a notched beam	Monotonic axial compression
Key measured parameters	Stress–strain curve, yield strength, Young’s modulus, ultimate tensile strength, elongation, reduction of area, fracture strain, post-necking behavior	Fracture toughness K_Ic_, J_Ic_, CTOD, CMOD, LLD, crack growth rate, reference temperature T_0_	Steel: CTOD, CMOD, LLD, J_Ic_, Concrete: Fracture energy G_F_, load–CMOD curve, load– deflection curve, tension softening curve	Compressive strength, elastic modulus, strain at peak stress, post-peak softening, fracture energy, Poisson’s ratio
Damage model inputs	Elastic–plastic constitutive law, stress–strain curve for the constitutive model calibration, stress triaxiality and Lode angle parameters for ductile damage models	Fracture toughness parameters for linear elastic and elastic–plastic fracture mechanics, Paris equation constants C and m for fatigue crack growth, reference temperature T_0_	Steel: Fracture toughness for elastic–plastic fracture mechanics Concrete: Fracture energy G_F_ and tension softening curve for phase-field and cohesive zone model calibration	Compressive strength, elastic modulus, and post-peak softening for constitutive damage model calibration
Key standards	ASTM E8/E8M, ASTM E21, ISO 6892-1, ISO 6892-2, ISO 12106, ISO 1099, ASTM E606/E606M, ASTM E466, ASTM A370, EN 10164	ASTM E399, ISO 12737, ASTM E1820, ISO 12135, ASTM E647, ASTM E2472, ASTM E1921	ASTM E399, ASTM E1820, ISO 12135, ASTM C1550, ASTM C1609, EN 14651, RILEM TC 50 FMC	ASTM C39, ASTM C469/C469M, EN 12390 3, ISO 1920 4, RILEM TC Recommendations
Beyond-standard advances	Miniaturized specimens, non-standard geometries for stress triaxiality and Lode angle, DIC-based virtual extensometry, Gleeble thermo-mechanical testing	Mini C(T) for reactor steels, modified C(T) for pipes, cross-weld specimens, side-grooved specimens	Mini SEN(B), wedge-splitting test (WST), wide plate (WP) specimens, size effect studies	Non-standard slenderness ratios, boundary condition studies, cyclic compression, fiber-reinforced variants

**Table 2:** Synthesis of experimental procedures: Advantages, limitations, and application scenarios.

Test Type	Uniaxial Tensile—UT	Compact Tension—C(T)	Single Edge Notched Bending—SEN(B)	Uniaxial Compression—UC
Material	Steel (primary)	Steel	Steel & Concrete	Concrete (primary)
Choose when	Baseline material characterization is needed. Also, for the constitutive law calibration, anisotropy, and forming behavior studies.	Structure contains or may contain a crack. Best suited for the integrity assessment of high-constraint, fracture-critical components like pipelines or pressure vessels.	Steel structures: Beams under bending-dominated loads. When fracture toughness of welded joints is needed across BM, WM, and HAZ zones. When CTOD or J-integral is required for damage model calibration Concrete structure: Quasi-brittle fracture characterization is required. Also, when fracture energy G_F_ is needed for damage model calibration.	Compressive strength and stiffness of concrete is needed. Used for dam, column, or structural element characterization.
Key advantages	Simple setup, widely standardized, compatible with DIC, strain gauges, and extensometers, availability of pre- and post-necking data. Also, applicable to a wide range of steel grades.	Direct output of fracture mechanics parameters, well- established standards. Applicable to fatigue and monotonic loading.	Applicable to both steel and concrete. Monitoring of stable crack propagation. Directly provides G_F_ for concrete damage models.	Simple setup, directly measures dominant loading mode in concrete structures, testing of field-sampled cores.
Key limitation	Does not capture crack propagation or fracture toughness; post-necking behavior needs correction methods (Bridgman, FEMU).	Expensive machining requires fatigue pre-cracking, sensitive to specimen thickness (plane strain validity).	Results are sensitive to specimen size, notch depth, span-to-depth ratio, and boundary conditions.	Boundary friction significantly affects results, size effect is debated. Limited to compressive loading.
Beyond-standard advances	Miniaturized specimens for limited material, non-standard geometries for triaxiality, elevated temperature testing	Miniaturized C(T) for irradiated reactor steels, modified geometries for pipe and weld assessment	Wedge-splitting test for large specimens, wide plate for low- constraint structures, size effect correction methods	Non-standard slenderness for column simulation, cyclic loading for fatigue damage, fiber-reinforced concrete testing
Typical application	Structural steel analysis, automotive steel forming, WAAM material testing, fatigue life assessment	Fitness-for-service assessment, reactor pressure vessel monitoring, pipeline fracture prediction	Dam monitoring, bridge and LNG tank assessment, concrete mix design, fiber- reinforced concrete characterization	Dam body study, column retrofit assessment, high-strength and fiber-reinforced concrete qualification

**Table 3:** Globally adopted standards that are found in the reviewed papers.

Ref.	Designation	Title	Specimen
[35]	ISO 6892-1	Metallic materials—Tensile testing—Part 1: Method of test at room temperature	Dog-bone (flat/cylindrical)
[36]	ASTM E8/E8M	Standard Test Methods for Tension Testing of Metallic Materials	Dog-bone (flat/cylindrical)
[43]	ASTM E21	Standard Test Methods for Elevated Temperature Tension Tests of Metallic Materials	Dog-bone (flat/cylindrical)
[55]	ISO 6892-2	Metallic materials—Tensile testing—Part 2: Method of test at elevated temperature	Dog-bone (flat/cylindrical)
[39]	ASTM A370	Standard Test Methods and Definitions for Mechanical Testing of Steel Products	Dog-bone (flat)
[49]	ISO 16630	Metallic materials—Sheet and strip—Hole expanding test	Flat sheet specimens
[67]	ISO 12106	Metallic materials—Fatigue testing—Axial strain-controlled method	Dog-bone (flat/cylindrical)
[68]	ISO 1099	Metallic materials—Fatigue testing—Axial force-controlled method	Dog-bone (flat/cylindrical)
[69]	ASTM E606/E606M	Standard Test Method for Strain-Controlled Fatigue Testing	Dog-bone (flat/cylindrical)
[70]	ASTM E466	Standard Practice for Conducting Force-Controlled Constant Amplitude Axial Fatigue Tests of Metallic Materials	Dog-bone (flat/cylindrical)
[86]	ASTM E399	Standard Test Method for Linear-Elastic Plane-Strain Fracture Toughness KIc of Metallic Materials	C(T), SEN(B)
[87]	ASTM E647	Standard Test Method for Measurement of Fatigue Crack Growth Rates	C(T)
[88]	ASTM E1820	Standard Test Method for Measurement of Fracture Toughness	C(T), SEN(B)
[89]	ASTM E2472	Standard Test Method for Determination of Resistance to Stable Crack Extension under Low-Constraint Conditions	C(T)
[90]	ASTM E1921	Standard Test Method for Determination of Reference Temperature, T_0_, for Ferritic Steels in the Transition Range	C(T)
[91]	ISO 12737	Metallic materials—Determination of plane-strain fracture toughness	C(T)
[92]	ISO 12135	Metallic materials—Unified method of test for the determination of quasi-static fracture toughness	C(T), SEN(B)
[117]	ISO 15653	Metallic materials—Method of test for the determination of quasi-static fracture toughness of welds	SEN(B)
[118]	ASTM C1550	Standard Test Method for Flexural Toughness of Fiber Reinforced Concrete (Using Centrally Loaded Round Panel)	Concrete panel
[119]	ASTM C1609	Standard Test Method for Flexural Performance of Fiber-Reinforced Concrete (Using Beam with Third-Point Loading)	Concrete beam
[120]	EN 14651	Test method for metallic fibered concrete—Measuring the flexural tensile strength (limit of proportionality (LOP), residual)	Concrete beam
[121]	RILEM TC-50 FMC	Recommendation for fracture mechanics of concrete	Concrete beam, SEN(B), WST
[122]	BS8571	Method of test for determination of fracture toughness in metallic materials using single edge notched tension (SENT) specimens	SENT
[127]	BS 7448	Fracture mechanics toughness tests Part 1. Method for determination of KIc, critical CTOD and critical J values of metallic materials	SEN(B)
[128]	WES 1108	Standard test method for Crack-Tip Opening Displacement (CTOD) fracture toughness measurement	SEN(B)
[135]	ISO 27306	Metallic materials—Method of constraint loss correction of CTOD fracture toughness for fracture assessment of steel components	SEN(B), Wide Plate
[146]	EN 1992-1-1	Eurocode 2: Design of concrete structures	Concrete grades (C30/37 etc.)
[143]	ASTM C33/C33M	Standard Specification for Concrete Aggregates	Concrete mix preparation
[148]	ASTM C78/C78M	Standard Test Method for Flexural Strength of Concrete (Using Simple Beam with Third-Point Loading)	Concrete beam
[152]	ASTM C39/C39M	Standard Method of Test for Compressive Strength of Cylindrical Concrete Specimens	Concrete cylinder
[153]	ASTM C469/C469M	Standard Test Method for Static Modulus of Elasticity and Poisson’s Ratio of Concrete in Compression	Concrete cylinder
[154]	EN 12390-3	Testing of hardened concrete—Part 3: Compressive strength of test specimens	Concrete cylinder/prism/cube
[155]	ISO 1920-4	Testing of concrete—Part 4: Strength of hardened concrete	Concrete cylinder/prism/cube

## Data Availability

No new data were created or analyzed in this study. Data sharing is not applicable to this article.
